# Descriptive norms caused increases in mask wearing during the COVID-19 pandemic

**DOI:** 10.1038/s41598-023-38593-w

**Published:** 2023-07-22

**Authors:** Samantha L. Heiman, Scott Claessens, Jessica D. Ayers, Diego Guevara Beltrán, Andrew Van Horn, Edward R. Hirt, Athena Aktipis, Peter M. Todd

**Affiliations:** 1 Department of Psychological and Brain Sciences, Indiana University Bloomington, 1101 E 10th St, Bloomington, IN 47405 USA; 2 School of Psychology, University of Auckland, Auckland, New Zealand; 3 Department of Psychological Science, Boise State University, Boise, ID USA; 4 Department of Psychology, Arizona State University, Tempe, AZ USA; 5 Department of Physics, Case Western Reserve University, Cleveland, OH USA; 6 Department of Art History, Case Western Reserve University, Cleveland, OH USA; 7 Cognitive Science Program, Indiana University Bloomington, Bloomington, IN USA

**Keywords:** Human behaviour, Social evolution

## Abstract

Human sociality is governed by two types of social norms: injunctive norms, which prescribe what people *ought* to do, and descriptive norms, which reflect what people *actually* do. The process by which these norms emerge and their causal influences on cooperative behavior over time are not well understood. Here, we study these questions through social norms influencing mask wearing during the COVID-19 pandemic. Leveraging 2 years of data from the United States (18 time points; *n* = 915), we tracked mask wearing and perceived injunctive and descriptive mask wearing norms as the pandemic unfolded. Longitudinal trends suggested that norms and behavior were tightly coupled, changing quickly in response to public health recommendations. In addition, longitudinal modeling revealed that descriptive norms caused future increases in mask wearing across multiple waves of data collection. These cross-lagged causal effects of descriptive norms were large, even after controlling for non-social beliefs and demographic variables. Injunctive norms, by contrast, had less frequent and generally weaker causal effects on future mask wearing. During uncertain times, cooperative behavior is more strongly driven by what others are actually doing, rather than what others think ought to be done.

## Introduction

Social norms are a key aspect of human sociality^1–3^. Broadly, social norms are defined as commonly known behavioral guidelines enforced by groups of people^4^. By coordinating the behavior of many individuals, social norms enable human groups to cooperate in the face of group-wide challenges and threats, such as resource scarcity, natural disasters, and infectious diseases^5^. Social norms are thus hypothesized to have played a key role in the evolution of large-scale cooperation in humans^6^.

Previous research has distinguished between two types of social norms: injunctive norms and descriptive norms^1,2,7^. Injunctive norms indicate what others tend to approve or disapprove of and often involve social sanctions if violated. By contrast, descriptive norms simply describe what most people are doing in a given situation, but carry no prescriptive information per se. According to the focus theory of normative conduct^2^, these two kinds of social norms often align, but they can also be in conflict with one another and differentially affect behavior depending on which norm is more salient. For example, there may be an injunctive norm that cleaning up litter at a picnic site is the right thing to do: one *ought* to behave this way. However, if an individual observes that most people are leaving their litter behind at the site, they may instead follow the descriptive norm and litter themselves.

Despite decades of research on injunctive and descriptive norms^2,8,9^, open questions remain regarding the emergence and causal influence of social norms^4,10^. First, how do injunctive and descriptive norms emerge over time within a population? Second, how do evolving injunctive and descriptive norms causally influence behavior over time?

Research has investigated how social norms emerge in a population over time. In the long term, cultural evolutionary models show that injunctive social norms can be vertically transmitted across generations by imitation or teaching, or horizontally diffused from neighboring populations^6^. However, less is known about how social norms arise endogenously within populations in the short term. While researchers have simulated the emergence of descriptive norms^11,12^, this modeling work does not capture how descriptive norms develop alongside injunctive norms in real-world settings. Recent work in behavioral economics has also suggested that injunctive norms of public good provisioning develop in tandem with cooperative behavior through repeated interactions^13^. But it remains unclear whether these findings generalize beyond the laboratory to real human populations.

With regards to normative influences on behavior, studies have demonstrated positive effects of descriptive norms on a variety of cooperative behaviors, including recycling^14^, paying taxes^15^, and sustainably reusing towels in hotels^16^. However, these studies have two key aspects that limit their ability to assess the causal impact of norms, both of which we address in our current work. First, studies have not accounted for other potential non-social explanations for behavior, such as perceptions of the effectiveness of the behavior and personal beliefs that one should behave in a certain way. These non-social beliefs, labeled “factual beliefs” and “personal normative beliefs”^17^, often correlate with descriptive and injunctive norms, but they are fundamentally different because they can cause behavior separately from social expectations about what others do or think should be done. For example, willingness to recycle might be driven by perceptions that recycling has a positive impact on the environment and/or personal beliefs that recycling is the right thing to do, even if social norms actively discourage recycling (e.g., recycling is not a common or socially approved behavior). It is thus important to control for non-social beliefs in studies of social norms, especially considering that personal norms have previously been shown to influence prosocial behavior^18,19^. Second, studies have tended to follow cross-sectional experimental designs in which social norm perceptions are manipulated by the researchers. However, social norms are not static: they change dynamically over time through processes of deliberation and interaction^20^. An alternative but underutilized method of assessing causality between social norms and cooperative behavior, while retaining ecological validity, is to follow these variables over time amidst a real, unfolding social dilemma.

To understand how social norms emerge over time and shape cooperative behavior in a non-experimental setting, we focus on mask wearing in the United States during the COVID-19 pandemic. In April 2020, one month after the World Health Organization declared COVID-19 a global pandemic, mask wearing was officially recommended by the Centers for Disease Control and Prevention (CDC) to minimize the spread of the disease^21^. Mask wearing has individual benefits, but the CDC also emphasized the collective benefits in reducing disease spread^22^. Indeed, mask wearing posed a social dilemma to many individuals, in that it imposed personal costs (e.g., difficulty breathing, disrupted social interaction) for the benefit of the community (e.g., “flattening the curve” to protect at-risk individuals). Thus, the development of mask wearing during the COVID-19 pandemic enables us to study the emergence of social norms and their causal effects on cooperative behavior over a short timescale within a single population.

Recent research has studied the relationships between social norms and protective COVID-19 behaviors. In the United States, one study found that perceptions of injunctive norms positively predicted intentions to stay at home to minimize exposure^23^, and another study found that experimentally-induced descriptive norms increased mask wearing intentions^24^. In Italy, an experimental study found no effect of messages highlighting descriptive norms, injunctive norms, or personal norms on time spent reading information about COVID-19 governmental rules^25^. In Germany, a two-wave study found that perceptions of descriptive norms positively predicted future protective behaviors, such as physical distancing^26^. These studies are informative, but since they are cross-sectional or only minimally longitudinal, they do not have the temporal granularity to capture fluctuating changes in norm strength and adherence across the pandemic. Furthermore, several of the studies do not control for potential confounding variables, such as demographics and political ideology. These variables are important to account for as they have previously been shown to be related to COVID-19 attitudes and behaviors^27,28^.

Here, we use 2 years of data from a representative sample of adults in the United States (18 time points; *n* = 915) to track the development of descriptive and injunctive mask wearing norms and mask wearing behavior over the course of the COVID-19 pandemic. Participants reported their frequency of mask wearing during in-person interactions, as well as their perceptions of descriptive and injunctive mask wearing norms. We also asked participants about their non-social mask wearing beliefs, demographics, and political ideology, and controlled for these factors. We used these longitudinal data to answer two main research questions in a specific real-world context. First, how do descriptive and injunctive mask wearing norms emerge over time? Second, how do descriptive and injunctive mask wearing norms causally influence mask wearing?

## Results

**Figure 1:**
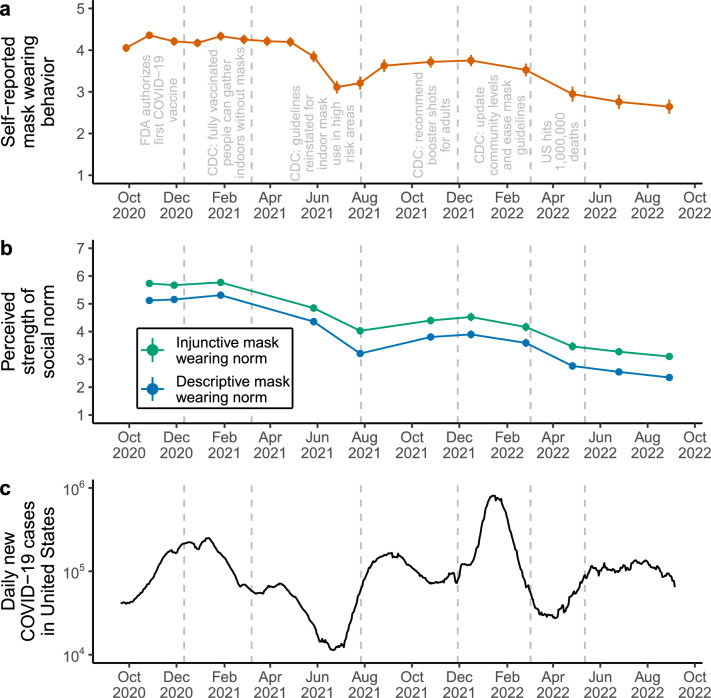
Timeline of self-reported mask wearing and perceived social norms in the United States during the COVID-19 pandemic. (**a**) Points and line ranges indicate means ± two standard errors for the self-reported mask wearing item. This item was measured across all 18 time points on a 5-point Likert scale, with higher values indicating increased frequency of personal mask wearing during in-person interactions. (**b**) Points and line ranges indicate means ± two standard errors for perceived injunctive mask wearing norms (green) and perceived descriptive mask wearing norms (blue). These items were measured across eleven time points on a 7-point Likert scale, with higher values indicating stronger perceived social norms. (**c**) Smoothed data for daily new COVID-19 cases in the United States, displayed on the log scale (data retrieved from Our World in Data; https://ourworldindata.org/). Across all panels, gray dashed lines represent significant pandemic-related events in the United States, such as vaccine approval from the Food and Drug Administration (FDA) and public health recommendations from the Centers for Disease Control and Prevention (CDC).

To answer our first research question about the emergence of mask wearing norms, we first visualized the average descriptive trends of self-reported norm perceptions across the entire study duration, showing how mask wearing social norms emerged and fluctuated over the course of the COVID-19 pandemic. Figure 1 plots self-reported mask wearing and perceptions of descriptive and injunctive mask wearing norms alongside relevant pandemic-related events in the United States, such as CDC public health recommendations and COVID-19 case numbers. These events were obtained from the CDC Museum’s COVID-19 Timeline^21^.

Two main observations can be made about the emergence and stability of social norms from these visualizations. First, social norms and behavior were tightly coupled over time. Although social norms are measured on fewer occasions than mask wearing, we can see that as mask wearing decreased in the summer of 2021, so too did perceived descriptive and injunctive mask wearing norms. Subsequently, the steep rise in COVID-19 case numbers in the fall of 2021 saw concomitant increases in both mask wearing and perceived social norms, before declining again in 2022. In line with these patterns, multilevel regression models revealed positive correlations between mask wearing and perceived descriptive mask wearing norms (*b* = 0.29, 95% confidence interval [0.23, 0.35], *p* < 0.001) and between mask wearing and perceived injunctive mask wearing norms (*b* = 0.26, 95% CI [0.22 0.30], *p* < .001) across individuals and time points (Supplementary Fig. S1; Supplementary Table S1).

Second, fluctuations in mask wearing and perceived social norms are in line with recommendations broadcasted by the CDC, an important institution governing public health in the United States. We do not have data for the very start of the pandemic in early 2020, but the high levels of mask wearing and strong perceived social norms at the start of our observation window likely emerged after the initial mask wearing recommendation from the CDC in April 2020. Perceived social norms and mask wearing subsequently declined after the CDC rescinded their mask wearing recommendation following widespread vaccine availability in March 2021, and then increased again after the CDC updated their guidelines for indoor mask use in high-risk areas in August 2021. Finally, perceived social norms and mask wearing declined again after the CDC eased mask wearing guidelines in March 2022. These trends were confirmed by a series of multilevel regression models with change points aligning with changes in CDC mask wearing recommendations (Supplementary Fig. S2; Supplementary Table S2).

Sample averages can provide informative trends, but they do not allow us to determine whether within-person changes in social norms caused future within-person changes in mask wearing over time. To answer our second research question about causal effects, we fitted a ten-wave random-intercept cross-lagged panel model^29,30^ to the longitudinal data. This model separately estimates stable trait-like between-person individual differences and within-person fluctuations from those trait levels for our main variables (self-reported mask wearing, perceived descriptive mask wearing norms, and perceived injunctive mask wearing norms) and control variables (factual beliefs and personal normative beliefs). In line with our proposed causal model (Supplementary Fig. S3), we also control for potential confounding in this model by including demographics (gender, age, ethnicity, socioeconomic status) and political orientation as exogenous controls.

Random-intercept cross-lagged panel models capture within-person changes over time with autoregressive and cross-lagged effects. Autoregressive effects represent “persistence” or “inertia” in within-person fluctuations from stable trait levels. In other words, a positive autoregressive effect indicates that being higher than average on one measure predicts being higher than average on that same measure in the following time point (this is not to be confused with the “stable trait level” over time, which is captured by the random intercepts in our model). For example, an autoregressive effect from mask wearing in February 2021 to future mask wearing in June 2021 would suggest that wearing masks more than average in February predicts wearing masks more than average in June. By contrast, and most relevant for the current study, cross-lagged effects represent the effect of a within-person fluctuation in one measure on future within-person fluctuations in other measures. In other words, a positive cross-lagged effect indicates that being higher than average on one measure predicts being higher than average on *another* measure in the following time point. For example, a cross-lagged effect from descriptive norms in February 2021 to future mask wearing in June 2021 would suggest that perceiving descriptive norms as stronger than average in February predicts wearing masks more than average in June. Cross-lagged effects are thus used to infer within-person causal influences over time. In what follows, we focus on the within-person autoregressive and cross-lagged effects for mask wearing and perceived social norms (see Supplementary Results for between-person results).

We first fitted a time-invariant model, which constrained the autoregressive effects, cross-lagged effects, covariances, and variances to equality over time. This model assumes that the relationships between variables are identical across all time points, giving us a sense of the average causal effects of social norms on mask wearing over time. According to established fit statistics, the time-invariant model fitted the data reasonably well (root mean square error of approximation (RMSEA) = 0.038, 95% CI [0.036, 0.040]; standardized root mean squared residual (SRMR) = 0.093; comparative fit index (CFI) = 0.906). Table 1 summarizes the autoregressive and cross-lagged parameters from the time-invariant model. Autoregressive effects were significantly positive for mask wearing, perceived descriptive norms, and perceived injunctive norms, indicating that being higher than average on these variables at time $t$ generally predicted being higher than average on the same variables at time $t + 1$. Crucially, cross-lagged effects from the time-invariant model revealed that perceived descriptive norms predicted future mask wearing (unstandardized *b* = 0.12, 95% CI [0.06 0.18], *p* < 0.001) while perceived injunctive norms did not (*b* = 0.00, 95% CI [− 0.05, 0.06], *p* = 0.893). In other words, perceiving descriptive norms as stronger than average at time $t$ generally predicted wearing masks more frequently than average at time $t + 1$, while no such effect existed for injunctive norms. All other cross-lagged effects between mask wearing, perceived descriptive norms, and perceived injunctive norms were significantly positive in the time-invariant model. This general pattern of results was unchanged when removing factual beliefs and personal normative beliefs from the time-invariant model (Supplementary Table S3) and when removing factual beliefs, personal normative beliefs, and all exogenous covariates from the time-invariant model (Supplementary Table S4).

**Table 1:** Unstandardized autoregressive and cross-lagged parameters from time-invariant random-intercept cross-lagged panel model.

Parameter	Estimate	SE	2.5%	97.5%	p
Mask wearing $\rightarrow$ Mask wearing	0.21	0.03	0.16	0.26	0.00
Mask wearing $\rightarrow$ Injunctive norms	0.04	0.02	0.00	0.07	0.04
Mask wearing $\rightarrow$ Descriptive norms	0.05	0.02	0.01	0.08	0.01
Injunctive norms $\rightarrow$ Mask wearing	0.00	0.03	− 0.05	0.06	0.89
Injunctive norms $\rightarrow$ Injunctive norms	0.27	0.03	0.21	0.32	0.00
Injunctive norms $\rightarrow$ Descriptive norms	0.12	0.02	0.07	0.17	0.00
Descriptive norms $\rightarrow$ Mask wearing	0.12	0.03	0.06	0.18	0.00
Descriptive norms $\rightarrow$ Injunctive norms	0.17	0.02	0.13	0.22	0.00
Descriptive norms $\rightarrow$ Descriptive norms	0.32	0.03	0.27	0.37	0.00

Arrows indicate the direction of prediction. Note that the effects of factual beliefs and personal normative beliefs are omitted from this table for clarity.

*SE* standard error.

Given that the strength of perceived social norms varied throughout our data collection window (Fig. 1), it is plausible that the causal effects of social norms on mask wearing may have changed over time as well, rather than being identical at each time point. To test whether the equality constraints over time were tenable^30^, we compared the time-invariant model to an alternative time-varying model that freely estimated the autoregressive effects, cross-lagged effects, covariances, and variances at each time point. This model assumes that the relationships between variables are different across different time points, allowing the causal effects of social norms on future mask wearing to vary over time. The time-varying model fitted the data better than the time-invariant model (RMSEA = 0.031, 95% CI [0.029, 0.033]; SRMR = 0.073; CFI = 0.955). Model comparison revealed that freely estimating the parameters over time resulted in improved model fit ($\Delta$AIC = -483.09, $\Delta \chi ^2$(320) = − 1123.09, *p* < 0.001) suggesting that there was substantial variability in the relationships between mask wearing and social norms across the course of the pandemic. Accordingly, we now turn to the results from the time-varying model.

**Figure 2:**
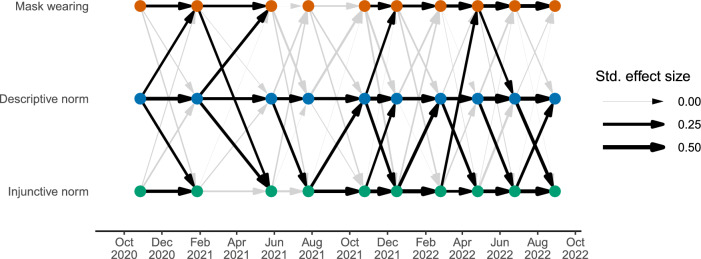
Path diagram of ten-wave time-varying random-intercept cross-lagged panel model. Circles represent data collection time points. Arrows represent within-person autoregressive effects (on one horizontal level) and cross-lagged effects (across levels) for mask wearing and perceived descriptive and injunctive norms, partitioning out stable between-person individual differences and controlling for factual beliefs, personal normative beliefs, demographics, and political orientation. Arrow thickness is scaled according to standardized effect size. Bolded arrows indicate significantly positive parameters, *p* < 0.05. Gray arrows indicate non-significant parameters.

**Figure 3:**
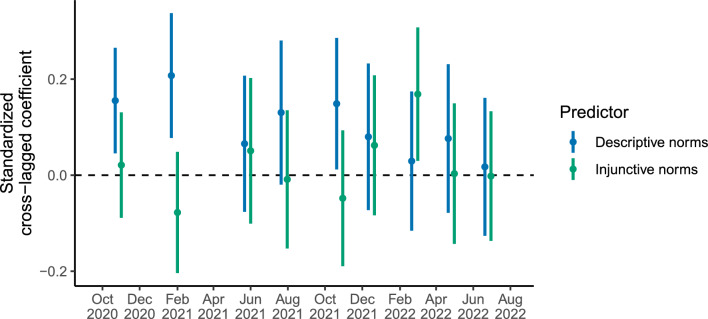
Standardized cross-lagged coefficients for descriptive norms and injunctive norms predicting future mask wearing in the ten-wave time-varying random-intercept cross-lagged panel model. Points are standardized estimates, lines are 95% confidence intervals.

Figure 2 displays the autoregressive and cross-lagged effects for social norms and mask wearing from the time-varying model (see Supplementary Table S5 for full list of estimated autoregressive and cross-lagged effects). In late 2020 and 2021, we find three occasions where within-person increases in perceived descriptive norms predicted future within-person increases in mask wearing. According to recent effect size guidelines for cross-lagged panel models^31^, the standardized coefficients for these cross-lagged effects were large (first wave, standardized $\beta$ = 0.16, *b* = 0.20, 95% CI [0.06, 0.34], *p* = .006; second wave, $\beta$ = 0.21, *b* = 0.26, 95% CI [0.10, 0.42], *p* = .002; fifth wave, $\beta$ = 0.15, *b* = 0.15, 95% CI [0.01, 0.29], *p* = 0.033). At other time points, the cross-lagged effects for descriptive norms tended to be estimated in a positive direction, though these estimates were not statistically significant and decreased in magnitude in 2022 (Fig. 3). Time-varying models without covariates revealed an additional significant cross-lagged effect of descriptive norms on future mask wearing at the fourth wave, but otherwise the general pattern of results for descriptive norms was unchanged (Supplementary Figs. S4–S7; Supplementary Tables S6 and S7).

In contrast, within-person increases in perceived injunctive norms only predicted future within-person increases in mask wearing at a single time point in March 2022 (seventh wave, $\beta$ = 0.17, *b* = 0.20, 95% CI [0.03, 0.37], *p* = 0.018). This cross-lagged effect was robust to the removal of covariates (Supplementary Figs. S4–S7, Supplementary Tables S6 and S7). At all other time points, the cross-lagged effects of injunctive norms on future mask wearing were non-significant and estimated to be generally weaker than the cross-lagged effects of descriptive norms (Fig. 3), explaining the lack of effect of injunctive norms in the time-invariant model.

One possible explanation for the generally weaker causal influence of injunctive norms on future mask wearing is that injunctive norms might only have been sufficiently salient in regions of the United States with stronger governmental enforcement of mask wearing. Previous work has shown that, during the pandemic, states with Democratic leadership tended to have more stringent mask wearing policies than states with Republican leadership^32^. It is thus plausible that injunctive norms had a stronger causal effect on future mask wearing in majority-Democrat states compared to majority-Republican states. To test this explanation, we fitted a multi-group time-invariant random-intercept cross-lagged panel model to the data, with separate groups for majority-Democrat and majority-Republican states. However, contrary to the salience account, perceived injunctive norms failed to predict future mask wearing in both Democrat states and Republican states (see Supplementary Results).

Beyond the effects of descriptive and injunctive norms on future mask wearing, our main time-varying model revealed other interesting patterns (Fig. 2). On a few occasions, we find evidence for a reciprocal relationship between social norms and mask wearing, whereby within-person increases in mask wearing predicted future within-person increases in perceived descriptive and injunctive norms. Moreover, several cross-lagged effects emerged between perceived descriptive and injunctive norms, demonstrating reciprocal within-person influences between these variables as the pandemic unfolded.

## Discussion

Using longitudinal data from the United States across 2 years of the COVID-19 pandemic, we aimed to understand how descriptive and injunctive mask wearing norms emerge and influence behavior in response to a naturally unfolding social dilemma. The trends of norm perceptions and self-reported mask wearing over time suggest that norms and behavior were tightly coupled and both changed dynamically in response to recommendations from public health authorities. Moreover, the results of our cross-lagged panel model indicate that descriptive norms caused future increases in mask wearing in the first year and a half of the pandemic. By contrast, injunctive norms were less frequently related to future mask wearing during the pandemic, with generally weaker effects than descriptive norms.

Our finding that social norms and mask wearing are tightly coupled over time provides real-world support for experimental evidence that social norms and cooperative behavior develop synergistically within groups via processes of social interaction^13^. The fact that these changes closely tracked the release of guidelines by the CDC supports the idea that institutions are part of the process by which culture and one’s own behaviors are mutually constructed^33^. Indeed, previous work has shown that formal institutions are critical for the emergence and rapid adoption of novel social norms^34^. While new norms can and do emerge spontaneously in populations, the process is slow compared to institution-driven norm change, which, as our trends have shown, can unfold over measurement intervals as short as 4 to 6 weeks.

We found that descriptive norms predicted future within-person increases in mask wearing, independent of the effects of injunctive norms, non-social beliefs, and demographic variables. This finding is in line with previous evidence showing that perceptions of descriptive norms were positively related to other protective COVID-19 behaviors^24,26^. There are several explanations for why descriptive norms have had these positive effects on protective COVID-19 behaviors like mask wearing. First, people may have followed descriptive norms to quickly coordinate their behavior with others during the pandemic. Descriptive norms are particularly useful for coordinating behavior during fast changing, threatening situations with a high degree of uncertainty, such as the COVID-19 pandemic^35^. This may help to explain why the effects of descriptive norms were more prevalent in earlier waves of our data, when uncertainty was highest. Second, people might have engaged in conditional cooperation, adapting their cooperation levels to the degree of cooperation in the population^36^. Descriptive mask wearing norms provide evidence that others are cooperating, increasing the likelihood that individuals will themselves contribute to the public good by wearing masks. Third, the increased frequency of mask wearing in the population might have created a bandwagon effect^37^, encouraging conformist copying. Under this view, people wear masks not to coordinate or cooperate, but simply because they see a majority of others engaging in the behavior. Future research will be required to determine the motivations underlying adherence to descriptive norms during uncertain times.

On the whole, we found that perceived injunctive norms tended not to predict future within-person increases in mask wearing, suggesting that injunctive norms and mask wearing were not strongly causally related during our data collection window. One possible explanation for this result is that, due to the increased opportunities to observe mask wearing in public, descriptive norms of mask wearing were more salient than injunctive norms during the pandemic. According to focus theory^2^, this difference in salience would produce behavior in line with descriptive norms and potentially suppress the effects of injunctive norms. By contrast, for more private behaviors like remaining indoors, it would have been less possible to observe other people’s behaviors, increasing the relative salience of injunctive norms. To test this idea, future research should expand our longitudinal approach to protective behaviors beyond mask wearing, including both public behaviors (e.g., physical distancing) and private behaviors (e.g., hand washing and home isolation). Nevertheless, it is worth noting that injunctive norms continued to have no overall causal effect on mask wearing when we focused specifically on majority-Democrat US states that tended to employ state-wide mask mandates^32^, suggesting that a lack of saliency cannot entirely explain why injunctive norms did not have a consistent effect on mask wearing during the pandemic.

Regarding injunctive mask wearing norms, several open questions remain. First, given that we see one time point in the later stages of the pandemic in which injunctive norms directly predicted future mask wearing, it is possible that there were also earlier time points where injunctive norms had effects on behavior. We do not have data from the very beginning of the pandemic (March–September 2020) to directly test this, but future work could test this by examining the effects of injunctive norms on behavior directly after the onset of a crisis. Second, it remains unclear how the source of the injunctive norm influences its efficacy. Our operationalization of injunctive norms referred in part to the behavior of respected others, and so the influence of the perceived approval of prestigious political and nationwide organizational leaders may be an important lever to test. In addition, given that we operationalized injunctive norms in part by local-level encouragement, this might explain why we see the only significant effect of injunctive norms in February 2022, as by this point the CDC was easing their nationwide mask guidelines. At this stage, people may have begun to look more to injunctive information from their local areas for encouragement on what was appropriate. Third, our longitudinal modeling showed that changes in perceived descriptive norms consistently influenced future changes in perceived injunctive norms throughout our study period, while the reverse was only true in the latter half of our study period. This suggests that initially changing empirical expectations of others’ behavior could potentially motivate compliance and shift later perceptions of injunctive norms^38^. Although our model was not specifically designed to address this question, future work should follow up on the temporal relationship between descriptive and injunctive norms with more fine-grained longitudinal data, perhaps experimentally intervening on perceptions of descriptive or injunctive norms to infer directions of causation.

It is unclear the extent to which our pattern of results might generalize to other cultural contexts. The United States is relatively close to the global average on cultural tightness-looseness, a cross-cultural dimension that measures nationwide strength of social norms^39,40^. We might therefore expect to see similar effects of social norms on mask wearing in countries with similar scores on this dimension. However, for countries with higher levels of cultural tightness than the United States, we might expect to see stronger effects of descriptive norms on mask wearing, additional effects of injunctive norms on mask wearing, and different dynamics between descriptive and injunctive norms. If borne out by the data, such patterns might explain why countries with higher levels of cultural tightness had fewer COVID-19 cases and deaths compared to culturally looser countries^40^. To test this, future research could expand our longitudinal approach to other cultural contexts.

Our results might not generalize to all social norms, behaviors, and social dilemmas. Norms governing sustainability in response to climate change, for example, might take longer to emerge, since the threat of climate change is more remote than the COVID-19 pandemic. For more distant social dilemmas that do not cause immediate day-to-day uncertainty, descriptive social norms may not necessarily drive cooperative behavior. Mask wearing is also a unique cooperative behavior in that it is not “purely” cooperative (i.e., imposing costs on the actor while providing benefits to targets). Much of past research on normative influences on prosocial behavior has focused on these purely unselfish cooperative behaviors^19^. Mask wearing is different as it does have individual benefits (e.g., reduced likelihood of contracting the disease). However, mask wearing also often imposes non-trivial costs on individuals (e.g., difficulty breathing, discomfort, disrupted social interaction) for the benefit of the wider group, meaning that it is still useful to conceptualize it as a prosocial or cooperative behavior. With these considerations, our results may be most pertinent for easily observable behaviors in response to an immediate or short-term threat that offer both benefits and costs to the individual in service to the wider group (e.g., other protective health behaviors, joining a protest or strike).

There are some limitations associated with our longitudinal survey design. First, we asked participants to self-report their frequency of mask wearing. However, self-report measures may be biased by anchoring effects and participants’ imperfect recall of their own behavior, resulting in measurement error^41^. Such measures were necessary to implement our 2-year longitudinal study, but future field studies of mask wearing norms could avoid this pitfall by directly measuring mask wearing through naturalistic observation. Second, as is common in longitudinal designs, there was substantial attrition over the course of the study (Supplementary Fig. S8). While this attrition did not substantially affect the demographic representativeness of our sample (Supplementary Fig. S9), the sample may have been self-selected in other unknown ways that introduced confounds. Future longitudinal work could encourage higher retention rates with more frequent reminders and fewer survey questions to maintain attention and interest.

Despite these caveats, we have shown that mask wearing norms developed rapidly in the United States population during the COVID-19 pandemic and tracked ongoing changes in both recommendations from authorities and current levels of mask wearing behavior. Moreover, we found that descriptive norms, rather than injunctive norms, were the main driver for future mask wearing. Importantly, this key finding slices two ways. Not only does it imply that higher local levels of mask wearing encouraged future personal mask use, but it also implies that *lower* local levels of mask wearing *discouraged* future personal mask use. This echoes recent reports of people in the United States not wanting to be “singled out” by being the only one wearing a mask in their community^42^. Organizations interested in combating such backfire effects might consider making masks easily and cheaply available in local settings (e.g., within smaller institutions or at events) and running media campaigns to show that mask wearing is common among “everyday people” doing “everyday things”. Once tested in target settings^43^, such interventions could leverage the power of consistent, visible community adherence to encourage protective behaviors in response to global pandemics like COVID-19.

## Materials and methods

### Ethical approval

All experimental protocols were approved by the Institutional Review Board of Arizona State University (STUDY00011678). All methods were carried out in accordance with relevant guidelines and regulations. All participants in this study provided informed consent.

### Participants and sampling

Using the platform Prolific (https://www.prolific.co/), we distributed surveys to a representative sample of adults from the United States (*n* = 915, *M*_age_ = 46 years, 75% White, 52% Women; see Supplementary Fig. S10 for geographic distribution). From September 2020 to October 2022, we asked participants to complete regular surveys of COVID-19 related attitudes and behaviors (for a visualization of all questions asked of this sample, see https://navigateobscurity.com/cooperation-conflict-lab/us-variable-lookup). This resulted in 18 unique time points of data collection during the pandemic. The first 12 time points were distributed monthly, while the remaining six time points were distributed every two months. Of the initial 915 participants, 634 returned to complete the survey at Time 2, while 347 participants continued through to Time 18 (see Supplementary Fig. S8 for attrition rates across all time points). However, this attrition did not substantially affect the demographic makeup of the sample through time (Supplementary Fig. S9). On average, participants were paid approximately $8 USD per hour for completing the surveys.

### Measures

#### Self-reported mask wearing

At every time point, participants were asked about the number of in-person interactions they had in the last 7 days and the last 24 hours. These two separate items specified that the interactions could have been either recreational or routine: “How many people (outside of your household) have you had in-person interactions with during the last [7 days/24 hours] (e.g., visiting with friends, buying food or supplies from a cashier)?” Following these questions, participants self-reported their mask wearing by answering: “During these in-person interactions, if you were closer than 6 feet (2 m) from the person(s) did you wear a face mask?” This question was asked regardless of how many in-person interactions the participants reported in the two interaction questions. Participants responded to the mask wearing question on a 5-point Likert scale, from Never (1) to Always (5). Responses to this question were weakly negatively associated with the number of in-person interactions that participants reported in the last 7 days and the last 24 hours, since wearing masks and social distancing are both COVID-19 protective behaviors (Supplementary Fig. S11).

#### Perceived descriptive and injunctive social norms

In 11 of the 18 time points (Time 2, 3, 5, 9, 11, 13, 14, 15, 16, 17, and 18), we asked questions about perceived descriptive and injunctive mask wearing norms.

Descriptive social norms were operationalized as the proportion of individuals in participants’ local areas wearing masks in routine and recreational settings. We measured perceived descriptive social norms as the mean of the following two items: “What proportion of people in your area wear a mask while doing routine activities indoors (e.g., running errands, shopping, going to work)?” and “What proportion of people in your area wear a mask while doing recreational/social activities indoors (e.g., going to the gym, eating at a restaurant, attending a party)?” These perceived descriptive social norm items were measured on 7-point Likert scales, from None (1) to All (7).

Injunctive social norms were operationalized as respected individuals wearing masks and community encouragement of mask wearing rules to emphasize the perceived social approval of the behavior from group leaders and the community at large. We measured perceived injunctive social norms as the mean of the following two items: “In general, how often do you see people that you respect and trust wearing a mask (e.g., on tv, news, etc.)?” and “How much are mask-wearing rules encouraged in your area (e.g., by local or state government officials, businesses, etc.)?” These perceived injunctive social norm items were measured on 7-point Likert scales, from Never/Rarely (1) to Very Often (7) for the first item, and from Strongly Discouraged (1) to Strongly Encouraged (7) for the second item.

There is potential overlap between these operationalizations of descriptive and injunctive norms. For example, the item asking how often participants see people that they respect and trust wearing masks could be capturing descriptive norms as well as injunctive norms. To address this concern about the construct validity of the four social norm items, at time point 7 we asked participants about their interpretations of the items. We asked participants whether each of the four items informed them about what people *are* doing or what people *should* be doing (i.e., giving descriptive or injunctive information). We found that participants rated the two descriptive norm questions as providing more descriptive information than the two injunctive norm questions, and vice versa, suggesting that the items are valid measures of perceived descriptive and injunctive social norms (see Supplementary Results and Supplementary Tables S8 and S9).

#### Additional control variables

To identify direct causal effects in our longitudinal analysis, we constructed a directed acyclic causal graph outlining the expected causal relationships between our variables (Supplementary Fig. S3). In this causal model, we included two kinds of non-social beliefs highlighted by previous research^17^: factual beliefs (i.e., beliefs about the effectiveness or consequences of mask wearing) and personal normative beliefs (i.e., personal beliefs about whether mask wearing is the right thing to do). These variables were included as potential mediators of the effects of descriptive and injunctive social norms on mask wearing. In addition, we also included demographics (gender, age, ethnicity, socioeconomic status, and political orientation) as common causes of all other variables. This is justified by evidence showing that these variables are associated with COVID-19 attitudes and behaviors^27,28^. Given this causal graph, it is necessary to control for factual beliefs, personal normative beliefs, and all demographic variables in order to estimate the direct causal effects of descriptive and injunctive norms on mask wearing behavior over time.

Non-social beliefs were measured in 12 of the 18 time points (Time 2, 4, 5, 7, 9, 11, 13, 14, 15, 16, 17, and 18). Factual beliefs were measured as the mean of the following two items: “I wear a face mask when going out in public to keep myself from getting sick” and “I wear a face mask when going out in public to prevent others from getting sick in case I may be infected but don’t know it yet”. Personal normative beliefs were measured with a single item: “Wearing a face mask when going out in public is the right thing to do”. These non-social belief items were measured on 7-point Likert scales, from Strongly Disagree (1) to Strongly Agree (7).

All demographic variables were measured at the first time point only. Gender was measured using a question that asked about participants’ biological sex (“What is your sex?”) with three possible categories (Male, Female, or Other). We are assuming within these analyses that participants identify as the gender associated with their biological sex. Age was measured numerically (“What is your age?”). Ethnicity was measured with a single item (“What is your ethnicity?”) with six possible categories (Asian or Pacific Islander, Hispanic or Latino/a, White or Caucasian, Black or African American, Native American, or Other). Socioeconomic status was operationalized through a composite measure averaging income, education level, and subjective socioeconomic status. Income was captured by a single item measured on a 9-point scale of increasing income amounts (“What was your combined household income in the previous year before taxes?”), education was captured by a single item measured on a 7-point scale of increasing education levels (“What is the highest level of education that you have completed?”), and subjective socioeconomic status was represented by a ladder with ten rungs, from those who are “worst off” at the bottom to those who are “best off” at the top. Political orientation was measured as the mean of the following two items: “How would you describe your political orientation with regard to social issues?” and “How would you describe your political orientation with regard to economic issues?”. These items were measured on 7-point Likert scales, from Very Liberal (1) to Very Conservative (7).

### Statistical analysis

To analyze average trends in self-reported mask wearing and perceived social norms, we fitted several multilevel regression models. First, to determine whether mask wearing and social norms were coupled over time, we regressed mask wearing on perceived descriptive and injunctive norms separately, including random intercepts and slopes for participants and time points. Second, to analyze whether changes over time were related to recommendations from the CDC, we regressed mask wearing and perceived social norms onto a continuous time predictor. These models included random intercepts and slopes for participants, as well as change points aligning with changes in CDC mask wearing recommendations. We estimated these multilevel regression models using the *lme4* R package^44^ and dealt with missing data via listwise deletion.

To quantify the within-person relationships between our variables over time, we fitted random-intercept cross-lagged panel models to our longitudinal data^29,30^. This structural equation model distinguishes between stable between-person trait levels and within-person fluctuations from trait levels. Positive cross-lagged effects from this model indicate that being above average on one variable at time $t$ predicts being above average in another variable at time $t + 1$. These models are considered the gold standard for identifying Granger causality in longitudinal datasets^29,30,45^.

We estimated the random-intercept cross-lagged panel models using the *lavaan* R package^46^. In line with our directed acyclic graph (Supplementary Fig. S3), we included three main variables (self-reported mask wearing, perceived descriptive norms, and perceived injunctive norms) and two control variables (factual beliefs and personal normative beliefs) in the model. For each of these variables, the model estimated a stable between-person trait level (random intercept) and time-specific within-person fluctuations from this trait level. We modeled autoregressive and cross-lagged effects between all five variables, and included gender, age, ethnicity, socioeconomic status, and political ideology as exogenous covariates. We restricted the analysis to the ten time points with available data for all five variables. We fitted both a time-invariant model (i.e., a model that constrained the within-person autoregressive effects, cross-lagged effects, covariances, and variance to equality over time) and a time-varying model that freely estimated all parameters. In both cases, full information maximum likelihood estimation was used to deal with missing data.

In addition to the full random-intercept cross-lagged panel model containing all covariates, we additionally fitted (1) a model with factual beliefs and personal normative beliefs removed and (2) a model with all covariates removed. We fitted both time-invariant and time-varying versions of these models. Finally, we fitted a time-invariant multi-group model that estimated parameters separately for majority-Democrat states and majority-Republican states, operationalized using the state-level results from the 2020 United States Presidential election. Results for these additional models are reported in the Supplementary Material.

Analyses were conducted retrospectively after data collection during the COVID-19 pandemic, meaning that no analyses were preregistered and all analyses should thus be considered exploratory. All analyses were conducted in R v4.1.1^47^. Visualizations were generated using the *cowplot*^48^ and *ggplot2*^49^ packages. The manuscript was reproducibly generated using the *targets*^50^ and *papaja*^51^ packages. All code and data are publicly available on GitHub: https://github.com/ScottClaessens/covidMaskWearing.

## Supplementary Information

Supplementary Information.

## Data Availability

All data and code to reproduce the statistical analyses in this manuscript are publicly available on GitHub: https://github.com/ScottClaessens/covidMaskWearing.
