# Supplementary material for: Descriptive norms caused increases in mask wearing during the COVID-19 pandemic
Source: Sci Rep. 2023 Jul 22;13:11856. doi: 10.1038/s41598-023-38593-w (PMC10363160; doi:10.1038/s41598-023-38593-w)
Supplement: Supplementary file 1 — Supplementary Information. [file 41598_2023_38593_MOESM1_ESM.pdf]

Supplementary Material

Descriptive norms caused increases in mask wearing during the COVID-19 pandemic

Samantha L. Heiman, Scott Claessens, Jessica D. Ayers, Diego Guevara Beltrán,
Andrew Van Horn, Edward R. Hirt, Athena Aktipis & Peter M. Todd

Supplementary Results

Construct validity for measures of perceived descriptive and injunctive norms. To evaluate the construct validity of our measures of perceived descriptive and injunctive norms, at Time 7 we asked participants to rate the extent to which each perceived norm item provided descriptive and injunctive information. For each item, participants were asked whether the item provided information about what people *are* doing, and whether the item provided information about what people *should* be doing. Participants responded on a 7-point Likert scale, from (1) Not At All to (7) Very Strongly. For a full list of questions, see Supplementary Table S8.

We fitted a multilevel model to determine whether participants rated the descriptive norm items as providing more descriptive information than the injunctive norm items, and vice versa. Pairwise comparisons from this model revealed that participants did differentiate the perceived norm items as expected (see Supplementary Table S9 for pairwise comparisons). Both descriptive norm items were seen as providing more descriptive information than both injunctive norm items. Conversely, both injunctive norm items were seen as providing more injunctive information than both descriptive norm items.

Between-person results from random-intercept cross-lagged panel model. The random-intercept cross-lagged panel model partitions the data into two components: (1) between-person trait-like individual differences that persist over time, and (2) within-person fluctuations from trait levels at individual time points. In the main text, we focused on the results from the within-person component of the model, specifically the autoregressive and cross-lagged parameters, as we were interested in within-person causal effects. However, the model also estimates the covariances between the random intercepts in the model, which can be interpreted as the correlations between between-person trait levels for each variable (i.e., whether people who are generally high on one variable tend to be generally high on another variable).

The covariances between the random intercepts in the full time-invariant model revealed positive correlations between stable trait levels of mask wearing and perceived social norms. On average across the whole study, participants who more frequently wore masks during in-person interactions also perceived stronger descriptive mask wearing norms (r = 0.16, 95% CI [0.01 0.30]) and stronger injunctive mask wearing norms (r = 0.30, 95% CI [0.17 0.43]). Stable trait perceptions of descriptive and injunctive mask wearing norms were also highly positively correlated (r = 0.68, 95% CI [0.61 0.75]). This general pattern of correlations at the between-person level was unchanged when removing all covariates and when unconstraining the within-person effects over time.

Multi-group random-intercept cross-lagged panel model. To test whether the causal effects of descriptive and injunctive norms varied across regions of the United States with different mask wearing policies, we fitted a multi-group random-intercept cross-lagged panel model. The model was fitted separately to two groups: majority-Democrat states and majority-Republican states. Participants were linked to US states via self-reported zip codes. We operationalised state-level partisanship using the state-level results for the 2020 Presidential election. For ease of interpretation, we constrained the within-person autoregressive effects, cross-lagged effects, covariances, and variances to equality over time. This multi-group model fitted the data well according to one fit statistic (RMSEA = 0.049, 95% CI [0.046 0.051]) but not according to others (SRMR = 0.100; CFI = 0.856).

In majority-Republican states, the main results are unchanged from the full model fitted to the whole sample: perceived descriptive norms predicted future mask wearing (b = 0.14, 95% CI [0.05 0.24], p = .004) while perceived injunctive norms did not (b = 0.00, 95% CI [-0.08 0.09], p = .910). In majority-Democrat states, perceived descriptive norms have a slightly weaker effect on future mask wearing (b = 0.08, 95% CI [0.00 0.17], p = .059) but the effect of perceived injunctive norms remains non-significant (b = -0.02, 95% CI [-0.11 0.06], p = .593). For full list of autoregressive and cross-lagged effects from the

multi-group model, see Supplementary Table S10.

Supplementary Figures

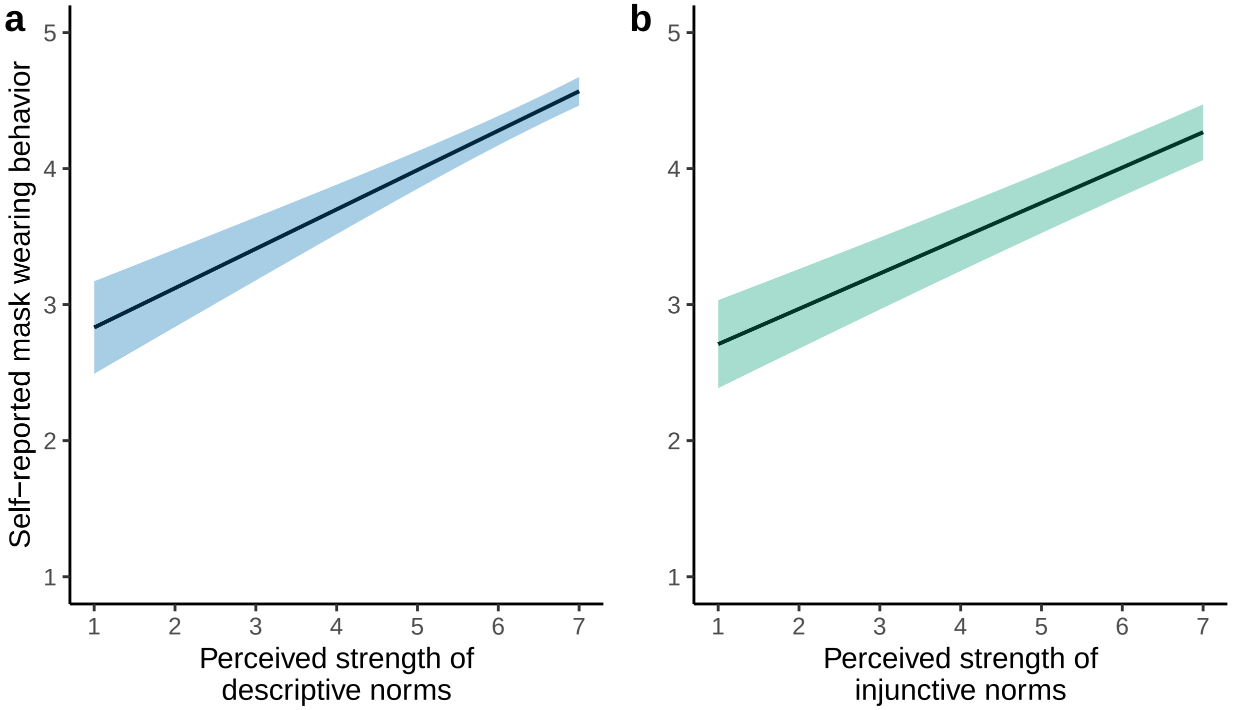

Figure S1. *Predictions from multilevel models with self-reported mask wearing as the outcome variable and (a) perceived strength of descriptive norms and (b) perceived strength of injunctive norms as independent predictor variables.* Models contain random intercepts for participants and time points. Lines are fixed effect regression lines from multilevel models, shaded areas are 95% confidence intervals.

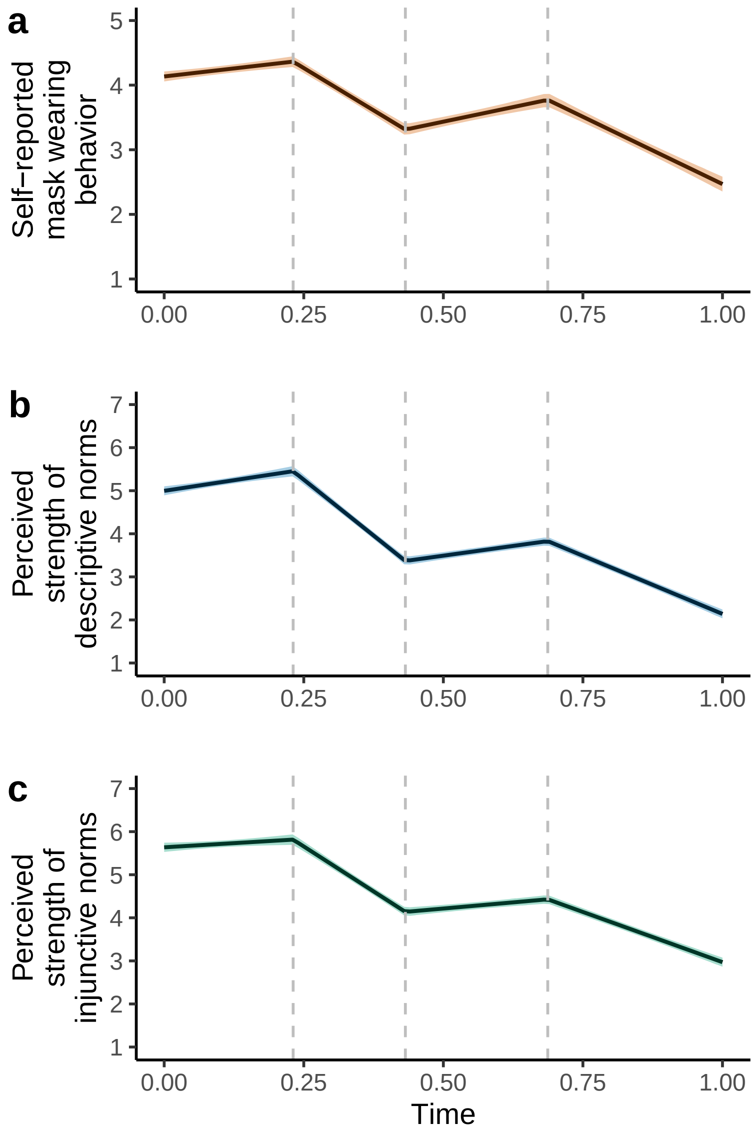

Figure S2. Predictions from multilevel models with change points in line with changes in CDC mask wearing recommendations. These models track temporal changes in (a) self-reported mask wearing, (b) perceived strength of descriptive norms, and (c) perceived strength of injunctive norms. Time is included as a continuous linear predictor, scaled between 0 and 1, with three forced change points (dashed lines). From the left, the first dashed line indicates when the CDC relaxed their mask wearing recommendations in March 2021, the second dashed line indicates when the CDC strengthened their mask wearing recommendations in July 2021, and the third dashed line indicates when the CDC updated their community levels and relaxed their mask wearing recommendations in March 2022. This results in the estimation of five fixed effect parameters: the initial intercept, the slope in the first window, the slope in the second window, the slope in the third window, and the slope is the fourth window. Bolded lines and shaded areas represent fixed effect regression lines from multilevel models and 95% confidence intervals, respectively.

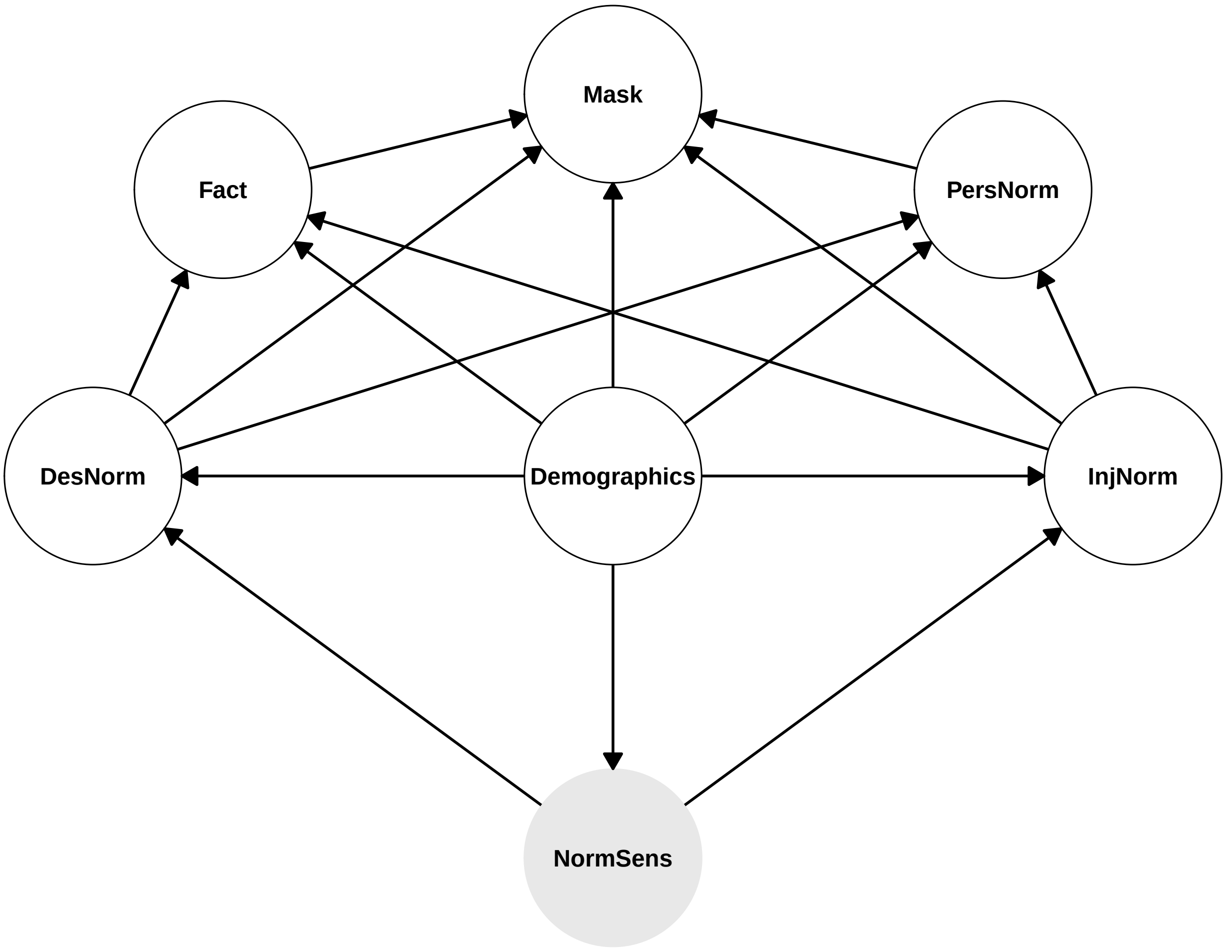

Figure S3. *Directed acyclic graph reflecting causal assumptions.* In this model, a general unobserved sensitivity to social norms (NormSens) causes perceptions of descriptive social norms (DesNorm) and perceptions of injunctive social norms (InjNorm), and perceptions of descriptive and injunctive norms directly cause mask wearing (Mask). Perceptions of descriptive and injunctive norms also indirectly cause mask wearing through non-social beliefs, specifically factual beliefs (Fact) and personal normative beliefs (PersNorm). Finally, demographic variables such as gender, age, ethnicity, socioeconomic status, and political orientation (summarised as Demographics) are exogenous variables that are common causes of all other variables. Using the backdoor criterion (Pearl, 1995), this causal model implies that it is necessary to control for perceptions of injunctive norms, factual beliefs, personal normative beliefs, and all demographics to estimate the direct causal effect of perceived descriptive norms on mask wearing. Similarly, it is necessary to control for perceptions of descriptive norms, factual beliefs, personal normative beliefs, and all demographics to estimate the direct causal effect of perceived injunctive norms on mask wearing.

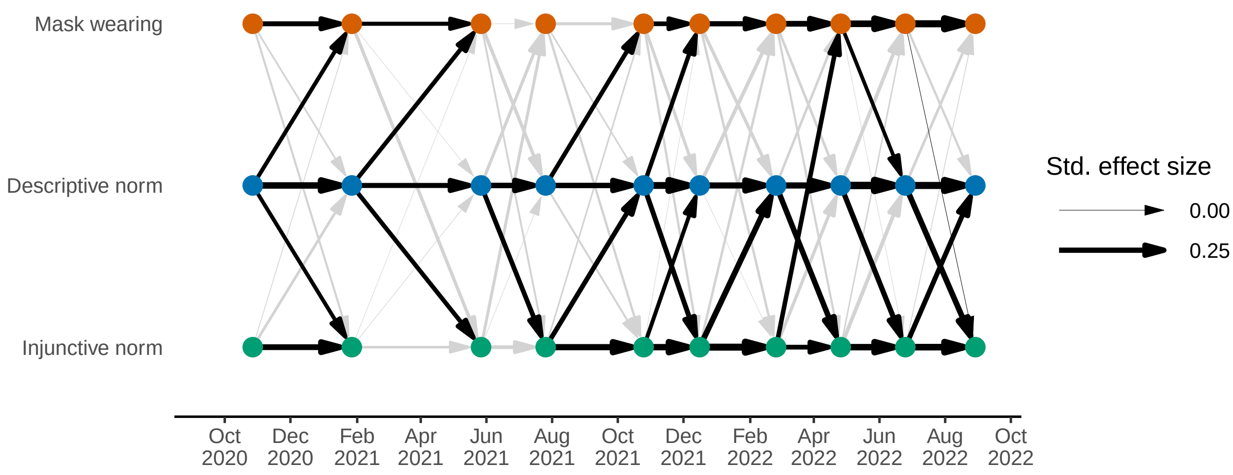

Figure S4. Path diagram of ten-wave time-varying random-intercept cross-lagged panel model (with factual beliefs and personal normative beliefs removed). Circles represent data collection time points. Arrows represent within-person autoregressive effects (on one horizontal level) and cross-lagged effects (across levels) for mask wearing and perceived descriptive and injunctive norms, partitioning out stable between-person individual differences and controlling for demographics and political orientation. Arrow thickness is scaled according to standardized effect size. Bolded arrows indicate significantly positive parameters, $p < 0.05$. Gray arrows indicate non-significant parameters.

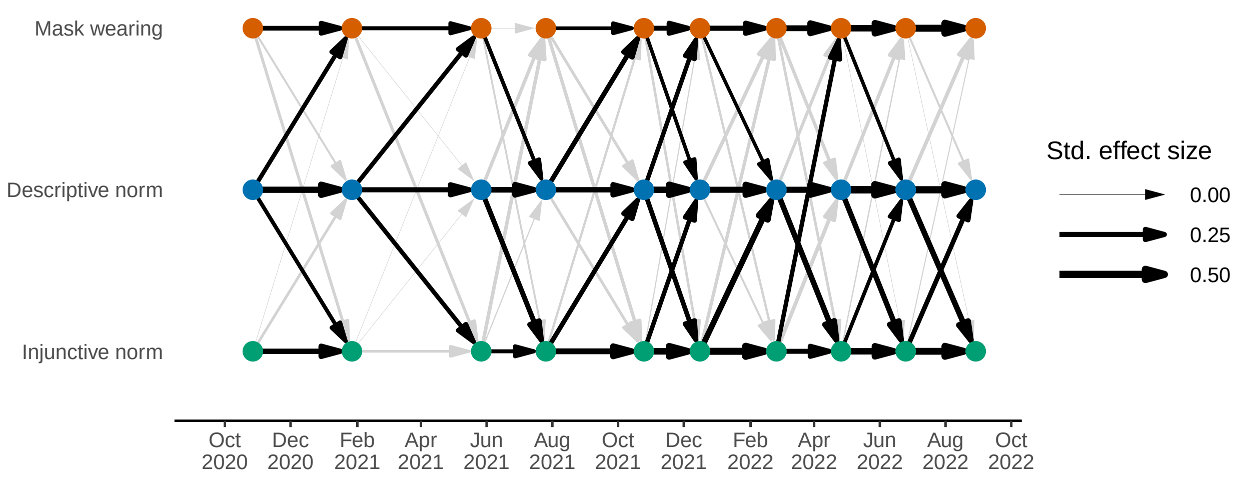

Figure S5. Path diagram of ten-wave time-varying random-intercept cross-lagged panel model (with factual beliefs, personal normative beliefs, and all exogenous covariates removed). Circles represent data collection time points. Arrows represent within-person autoregressive effects (on one horizontal level) and cross-lagged effects (across levels) for mask wearing and perceived descriptive and injunctive norms, partitioning out stable between-person individual differences. Arrow thickness is scaled according to standardized effect size. Bolded arrows indicate significantly positive parameters, $p < 0.05$. Gray arrows indicate non-significant parameters.

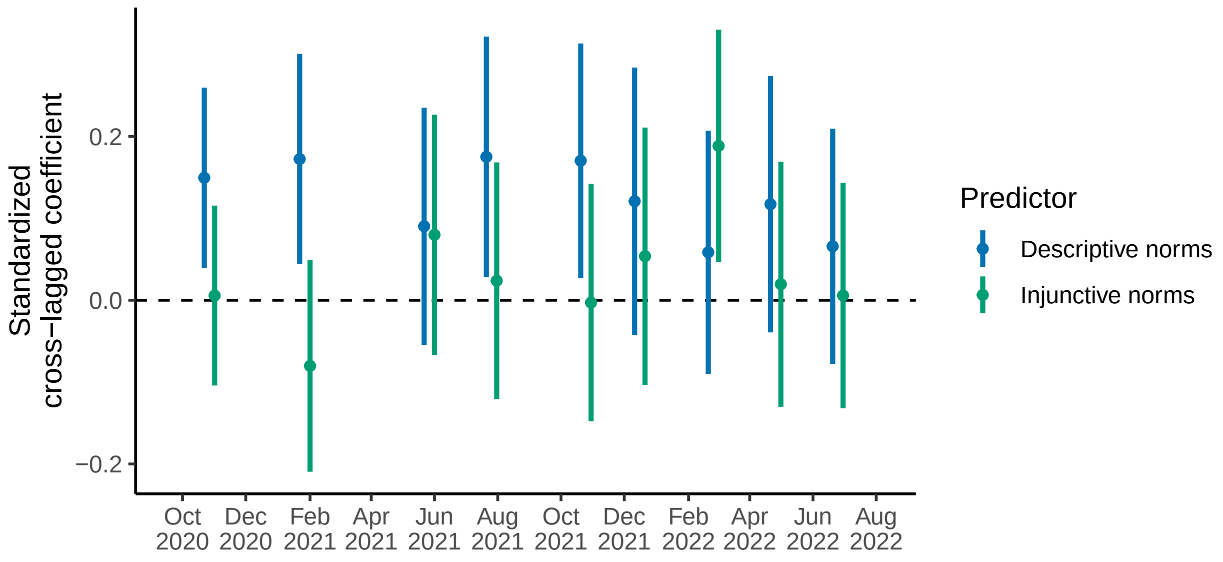

Figure S6. Standardized cross-lagged coefficients for descriptive norms and injunctive norms predicting future mask wearing in the ten-wave time-varying random-intercept cross-lagged panel model (with factual beliefs and personal normative beliefs removed). Points are standardized estimates, lines are 95% confidence intervals.

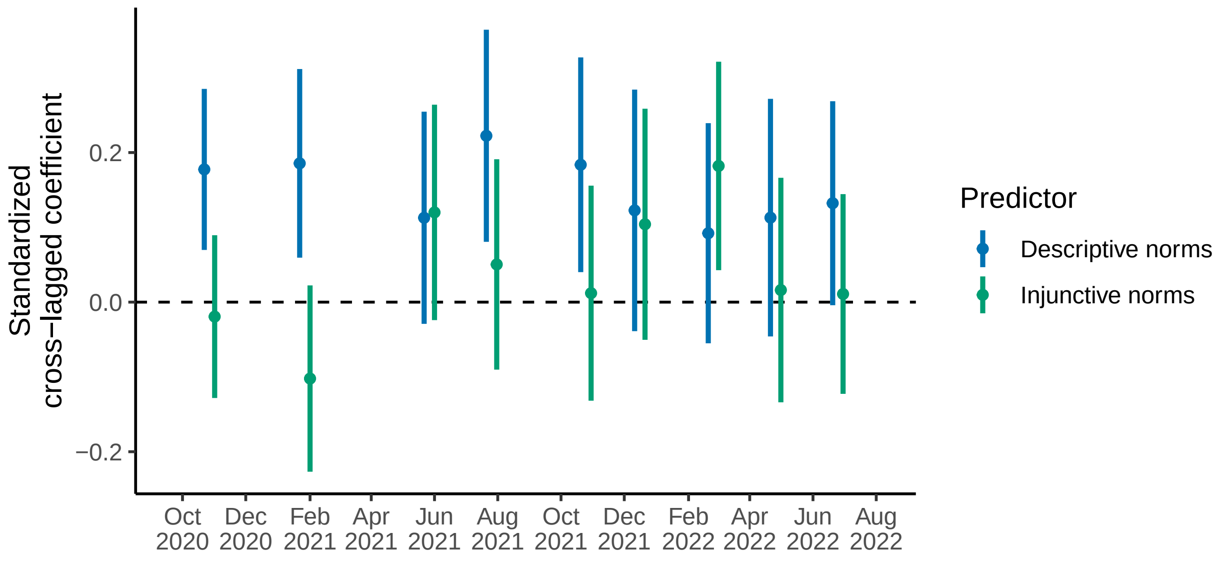

Figure S7. Standardized cross-lagged coefficients for descriptive norms and injunctive norms predicting future mask wearing in the ten-wave time-varying random-intercept cross-lagged panel model (with factual beliefs, personal normative beliefs, and all exogenous covariates removed). Points are standardized estimates, lines are 95% confidence intervals.

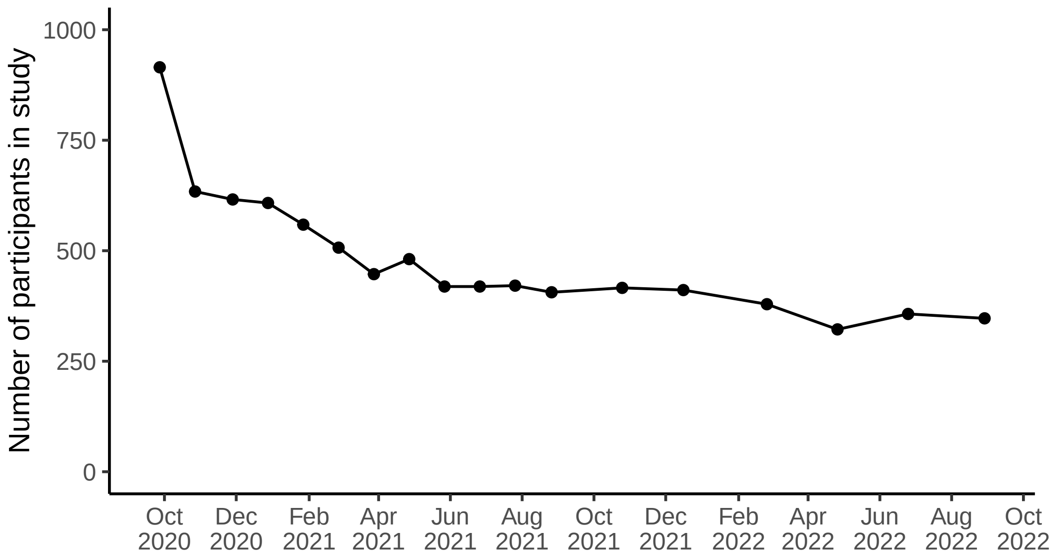

Figure S8. *Attrition across the course of the study.*

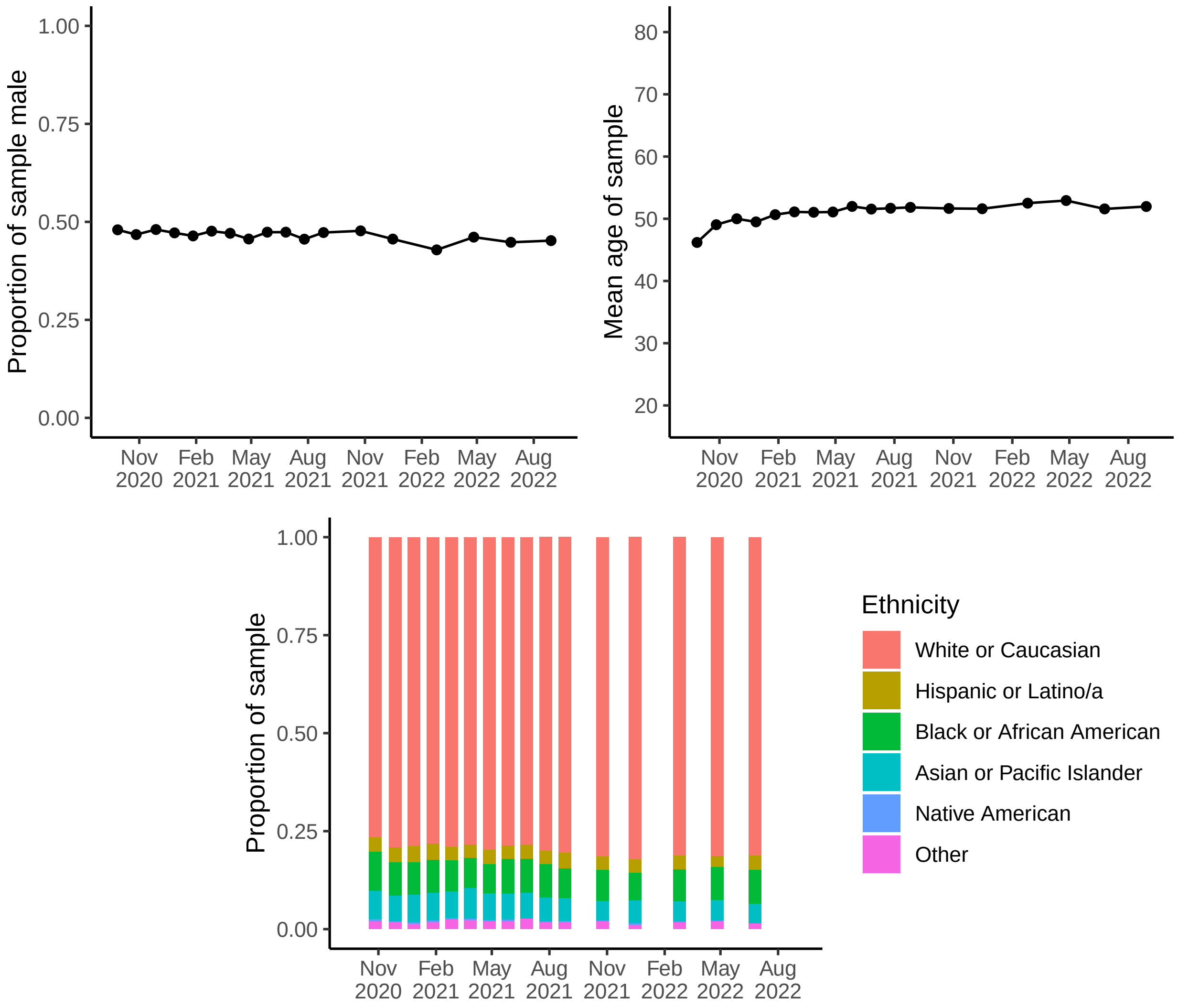

Figure S9. *Demographics of the sample across the course of the study.*

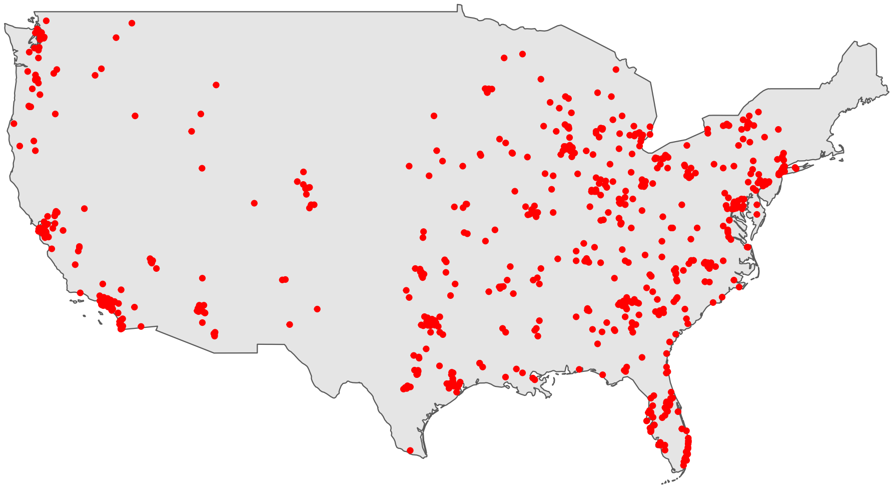

Figure S10. *Map of the United States with participant zip code locations.*

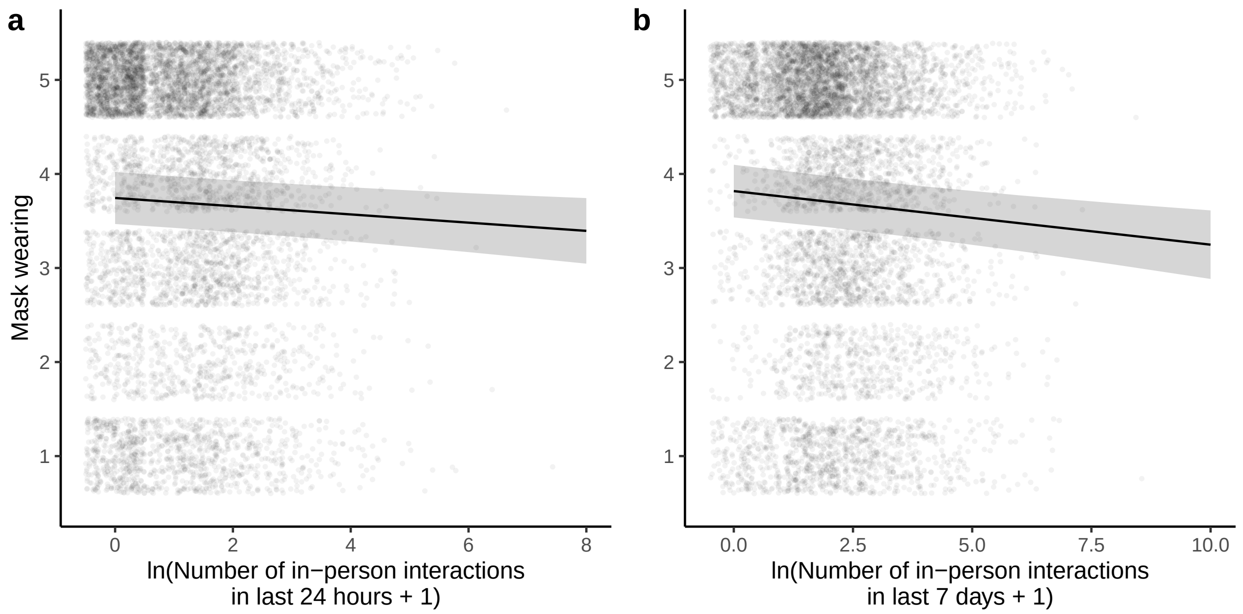

Figure S11. The relationship between self-reported mask wearing and the logged number of in-person interactions in the last 24 hours (a) and in the last 7 days (b). Lines and shaded areas are predictions and 95% confidence intervals from multilevel models with random intercepts for timepoint and participant. The first multilevel model revealed a negative relationship between mask wearing and the log number of in-person interactions in the last 24 hours ($b = -0.04$, $SE = 0.02$, $p = .007$). The second multilevel model revealed a negative relationship between mask wearing and the log number of in-person interactions in the last 7 days ($b = -0.06$, $SE = 0.02$, $p < .001$).

Supplementary Tables

Table S1

Unstandardized fixed effect parameters from multilevel models: perceptions of social norm strength predicting self-reported mask wearing. Standard errors are included in brackets.

	Model 1	Model 2
Intercept	2.54	2.45
	(0.20)	(0.18)
Descriptive norms	0.29	
	(0.03)	
Injunctive norms		0.26
		(0.02)
N	4785	4798
N (id)	783	783
N (time)	11	11
AIC	15309.62	15411.28
BIC	15367.88	15469.57
R2 (fixed)	0.10	0.08
R2 (total)	0.47	0.47

Table S2

Unstandardized fixed effect parameters from multilevel models: trends over time with change points at CDC events.

	Mask wearing	Descriptive norms	Injunctive norms
Intercept	4.13, 95% CI [4.05 4.21]	5.00, 95% CI [4.90 5.10]	5.64, 95% CI [5.53 5.74]
Slope1	0.99, 95% CI [0.56 1.42]	1.98, 95% CI [1.24 2.72]	0.78, 95% CI [0.03 1.52]
Slope2	-5.23, 95% CI [-5.73 -4.71]	-10.38, 95% CI [-11.07 -9.67]	-8.36, 95% CI [-9.05 -7.64]
Slope3	1.80, 95% CI [1.33 2.33]	1.82, 95% CI [1.35 2.25]	1.15, 95% CI [0.68 1.59]
Slope4	-4.16, 95% CI [-4.65 -3.68]	-5.40, 95% CI [-5.77 -4.99]	-4.66, 95% CI [-5.03 -4.25]
N	8505	4851	4861
R2 (fixed)	0.11	0.4	0.34
R2 (total)	0.38	0.68	0.67

Table S3

Unstandardized autoregressive and cross-lagged parameters from time-invariant random-intercept cross-lagged panel model (with factual beliefs and personal normative beliefs removed). Arrows indicate the direction of prediction.

Parameter	Estimate	SE	2.5%	97.5%	p
Mask wearing → Mask wearing	0.23	0.02	0.18	0.28	0.00
Mask wearing → Injunctive norms	0.02	0.02	-0.02	0.05	0.27
Mask wearing → Descriptive norms	0.04	0.02	0.01	0.08	0.02
Injunctive norms → Mask wearing	0.02	0.03	-0.04	0.08	0.48
Injunctive norms → Injunctive norms	0.26	0.03	0.21	0.31	0.00
Injunctive norms → Descriptive norms	0.12	0.02	0.07	0.17	0.00
Descriptive norms → Mask wearing	0.15	0.03	0.09	0.21	0.00
Descriptive norms → Injunctive norms	0.17	0.02	0.12	0.21	0.00
Descriptive norms → Descriptive norms	0.32	0.03	0.27	0.38	0.00

Table S4
Unstandardized autoregressive and cross-lagged parameters from time-invariant random-intercept cross-lagged panel model (with factual beliefs, personal normative beliefs, and all exogenous covariates removed). Arrows indicate the direction of prediction.

Parameter	Estimate	SE	2.5%	97.5%	p
Mask wearing → Mask wearing	0.25	0.02	0.20	0.30	0.00
Mask wearing → Injunctive norms	0.03	0.02	0.00	0.07	0.06
Mask wearing → Descriptive norms	0.07	0.02	0.03	0.10	0.00
Injunctive norms → Mask wearing	0.03	0.03	-0.03	0.09	0.32
Injunctive norms → Injunctive norms	0.28	0.03	0.23	0.33	0.00
Injunctive norms → Descriptive norms	0.14	0.02	0.09	0.18	0.00
Descriptive norms → Mask wearing	0.18	0.03	0.12	0.24	0.00
Descriptive norms → Injunctive norms	0.19	0.02	0.14	0.24	0.00
Descriptive norms → Descriptive norms	0.34	0.03	0.29	0.40	0.00

Table S5

Unstandardized autoregressive and cross-lagged parameters from time-varying random-intercept cross-lagged panel model (full model). Variable name suffixes indicate time points. Arrows indicate the direction of prediction.

Parameter	Estimate	SE	2.5%	97.5%	p
MaskWearing_02 → MaskWearing_05	0.24	0.06	0.12	0.36	0.00
MaskWearing_02 → InjunctiveNorms_05	0.04	0.05	-0.05	0.14	0.39
MaskWearing_02 → DescriptiveNorms_05	0.02	0.05	-0.08	0.12	0.64
MaskWearing_02 → FactualBeliefs_05	0.00	0.05	-0.10	0.09	0.97
MaskWearing_02 → PersonalBeliefs_05	-0.01	0.04	-0.10	0.07	0.74
MaskWearing_05 → MaskWearing_09	0.18	0.06	0.06	0.31	0.00
MaskWearing_05 → InjunctiveNorms_09	0.14	0.06	0.03	0.26	0.01
MaskWearing_05 → DescriptiveNorms_09	0.01	0.06	-0.12	0.13	0.92
MaskWearing_05 → FactualBeliefs_09	0.15	0.07	0.01	0.29	0.03
MaskWearing_05 → PersonalBeliefs_09	0.06	0.07	-0.07	0.19	0.35
MaskWearing_09 → MaskWearing_11	-0.04	0.08	-0.19	0.12	0.62
MaskWearing_09 → InjunctiveNorms_11	0.04	0.06	-0.08	0.16	0.51
MaskWearing_09 → DescriptiveNorms_11	0.10	0.06	-0.02	0.21	0.09
MaskWearing_09 → FactualBeliefs_11	0.22	0.08	0.07	0.37	0.00
MaskWearing_09 → PersonalBeliefs_11	0.15	0.06	0.02	0.27	0.02
MaskWearing_11 → MaskWearing_13	0.05	0.06	-0.06	0.17	0.36
MaskWearing_11 → InjunctiveNorms_13	0.04	0.05	-0.06	0.14	0.47
MaskWearing_11 → DescriptiveNorms_13	0.05	0.06	-0.06	0.16	0.38
MaskWearing_11 → FactualBeliefs_13	0.03	0.06	-0.08	0.15	0.55
MaskWearing_11 → PersonalBeliefs_13	0.03	0.05	-0.08	0.14	0.58
MaskWearing_13 → MaskWearing_14	0.19	0.06	0.06	0.31	0.00
MaskWearing_13 → InjunctiveNorms_14	0.08	0.05	-0.03	0.18	0.14
MaskWearing_13 → DescriptiveNorms_14	0.09	0.05	-0.01	0.20	0.08
MaskWearing_13 → FactualBeliefs_14	0.09	0.06	-0.02	0.21	0.12
MaskWearing_13 → PersonalBeliefs_14	0.01	0.05	-0.09	0.11	0.82
MaskWearing_14 → MaskWearing_15	0.22	0.06	0.10	0.35	0.00
MaskWearing_14 → InjunctiveNorms_15	0.06	0.06	-0.05	0.17	0.31
MaskWearing_14 → DescriptiveNorms_15	0.07	0.05	-0.03	0.17	0.18

Table S5 continued

Parameter	Estimate	SE	2.5%	97.5%	p
MaskWearing_14 → FactualBeliefs_15	0.05	0.06	-0.07	0.18	0.42
MaskWearing_14 → PersonalBeliefs_15	-0.07	0.06	-0.20	0.05	0.27
MaskWearing_15 → MaskWearing_16	0.29	0.08	0.14	0.44	0.00
MaskWearing_15 → InjunctiveNorms_16	0.00	0.06	-0.12	0.13	0.94
MaskWearing_15 → DescriptiveNorms_16	-0.01	0.06	-0.12	0.10	0.91
MaskWearing_15 → FactualBeliefs_16	0.09	0.08	-0.06	0.25	0.24
MaskWearing_15 → PersonalBeliefs_16	0.08	0.07	-0.06	0.22	0.24
MaskWearing_16 → MaskWearing_17	0.37	0.07	0.23	0.51	0.00
MaskWearing_16 → InjunctiveNorms_17	0.01	0.05	-0.08	0.10	0.83
MaskWearing_16 → DescriptiveNorms_17	0.10	0.04	0.01	0.18	0.02
MaskWearing_16 → FactualBeliefs_17	0.27	0.08	0.12	0.42	0.00
MaskWearing_16 → PersonalBeliefs_17	0.15	0.07	0.01	0.28	0.03
MaskWearing_17 → MaskWearing_18	0.38	0.06	0.26	0.50	0.00
MaskWearing_17 → InjunctiveNorms_18	-0.04	0.04	-0.11	0.04	0.32
MaskWearing_17 → DescriptiveNorms_18	0.02	0.03	-0.04	0.09	0.49
MaskWearing_17 → FactualBeliefs_18	0.13	0.07	0.00	0.26	0.05
MaskWearing_17 → PersonalBeliefs_18	0.06	0.06	-0.06	0.18	0.34
InjunctiveNorms_02 → MaskWearing_05	0.03	0.07	-0.11	0.17	0.71
InjunctiveNorms_02 → InjunctiveNorms_05	0.30	0.06	0.19	0.42	0.00
InjunctiveNorms_02 → DescriptiveNorms_05	0.07	0.06	-0.04	0.19	0.23
InjunctiveNorms_02 → FactualBeliefs_05	0.05	0.05	-0.06	0.16	0.37
InjunctiveNorms_02 → PersonalBeliefs_05	0.00	0.05	-0.10	0.10	0.97
InjunctiveNorms_05 → MaskWearing_09	-0.10	0.08	-0.26	0.06	0.23
InjunctiveNorms_05 → InjunctiveNorms_09	0.09	0.08	-0.07	0.24	0.26
InjunctiveNorms_05 → DescriptiveNorms_09	0.03	0.08	-0.12	0.19	0.68
InjunctiveNorms_05 → FactualBeliefs_09	0.03	0.09	-0.16	0.21	0.78
InjunctiveNorms_05 → PersonalBeliefs_09	-0.06	0.09	-0.23	0.11	0.51
InjunctiveNorms_09 → MaskWearing_11	0.07	0.10	-0.13	0.27	0.51
InjunctiveNorms_09 → InjunctiveNorms_11	0.13	0.08	-0.03	0.28	0.12
InjunctiveNorms_09 → DescriptiveNorms_11	-0.02	0.07	-0.17	0.12	0.77

Table S5 continued

Parameter	Estimate	SE	2.5%	97.5%	p
InjunctiveNorms_09 → FactualBeliefs_11	0.00	0.10	-0.20	0.19	0.98
InjunctiveNorms_09 → PersonalBeliefs_11	0.02	0.08	-0.14	0.19	0.76
InjunctiveNorms_11 → MaskWearing_13	-0.01	0.08	-0.16	0.14	0.91
InjunctiveNorms_11 → InjunctiveNorms_13	0.31	0.07	0.18	0.45	0.00
InjunctiveNorms_11 → DescriptiveNorms_13	0.24	0.07	0.10	0.38	0.00
InjunctiveNorms_11 → FactualBeliefs_13	0.18	0.08	0.03	0.33	0.02
InjunctiveNorms_11 → PersonalBeliefs_13	0.10	0.07	-0.04	0.23	0.18
InjunctiveNorms_13 → MaskWearing_14	-0.05	0.08	-0.21	0.11	0.51
InjunctiveNorms_13 → InjunctiveNorms_14	0.42	0.07	0.28	0.55	0.00
InjunctiveNorms_13 → DescriptiveNorms_14	0.16	0.07	0.02	0.30	0.02
InjunctiveNorms_13 → FactualBeliefs_14	0.03	0.08	-0.12	0.18	0.68
InjunctiveNorms_13 → PersonalBeliefs_14	0.10	0.07	-0.03	0.22	0.15
InjunctiveNorms_14 → MaskWearing_15	0.07	0.08	-0.09	0.23	0.40
InjunctiveNorms_14 → InjunctiveNorms_15	0.45	0.07	0.31	0.59	0.00
InjunctiveNorms_14 → DescriptiveNorms_15	0.28	0.07	0.16	0.41	0.00
InjunctiveNorms_14 → FactualBeliefs_15	0.11	0.08	-0.05	0.27	0.16
InjunctiveNorms_14 → PersonalBeliefs_15	0.07	0.08	-0.09	0.23	0.38
InjunctiveNorms_15 → MaskWearing_16	0.20	0.09	0.03	0.37	0.02
InjunctiveNorms_15 → InjunctiveNorms_16	0.19	0.07	0.05	0.33	0.01
InjunctiveNorms_15 → DescriptiveNorms_16	0.04	0.06	-0.09	0.16	0.56
InjunctiveNorms_15 → FactualBeliefs_16	0.04	0.09	-0.14	0.22	0.67
InjunctiveNorms_15 → PersonalBeliefs_16	0.12	0.08	-0.03	0.28	0.12
InjunctiveNorms_16 → MaskWearing_17	0.00	0.10	-0.19	0.20	0.97
InjunctiveNorms_16 → InjunctiveNorms_17	0.32	0.07	0.19	0.44	0.00
InjunctiveNorms_16 → DescriptiveNorms_17	0.10	0.06	-0.01	0.21	0.08
InjunctiveNorms_16 → FactualBeliefs_17	0.00	0.11	-0.21	0.21	0.97
InjunctiveNorms_16 → PersonalBeliefs_17	-0.04	0.09	-0.22	0.14	0.68
InjunctiveNorms_17 → MaskWearing_18	0.00	0.10	-0.21	0.20	0.98
InjunctiveNorms_17 → InjunctiveNorms_18	0.37	0.06	0.25	0.50	0.00
InjunctiveNorms_17 → DescriptiveNorms_18	0.18	0.06	0.07	0.29	0.00

Table S5 continued

Parameter	Estimate	SE	2.5%	97.5%	p
InjunctiveNorms_17 → FactualBeliefs_18	0.07	0.11	-0.15	0.30	0.53
InjunctiveNorms_17 → PersonalBeliefs_18	-0.02	0.10	-0.22	0.18	0.84
DescriptiveNorms_02 → MaskWearing_05	0.20	0.07	0.06	0.34	0.01
DescriptiveNorms_02 → InjunctiveNorms_05	0.18	0.06	0.07	0.30	0.00
DescriptiveNorms_02 → DescriptiveNorms_05	0.40	0.06	0.29	0.52	0.00
DescriptiveNorms_02 → FactualBeliefs_05	0.05	0.06	-0.06	0.16	0.39
DescriptiveNorms_02 → PersonalBeliefs_05	0.02	0.05	-0.08	0.12	0.71
DescriptiveNorms_05 → MaskWearing_09	0.26	0.08	0.10	0.42	0.00
DescriptiveNorms_05 → InjunctiveNorms_09	0.26	0.08	0.11	0.41	0.00
DescriptiveNorms_05 → DescriptiveNorms_09	0.29	0.08	0.13	0.45	0.00
DescriptiveNorms_05 → FactualBeliefs_09	0.19	0.09	0.01	0.37	0.04
DescriptiveNorms_05 → PersonalBeliefs_09	0.29	0.09	0.12	0.46	0.00
DescriptiveNorms_09 → MaskWearing_11	0.08	0.09	-0.09	0.25	0.36
DescriptiveNorms_09 → InjunctiveNorms_11	0.19	0.07	0.06	0.33	0.01
DescriptiveNorms_09 → DescriptiveNorms_11	0.24	0.07	0.11	0.37	0.00
DescriptiveNorms_09 → FactualBeliefs_11	0.05	0.09	-0.12	0.22	0.59
DescriptiveNorms_09 → PersonalBeliefs_11	0.07	0.07	-0.07	0.22	0.31
DescriptiveNorms_11 → MaskWearing_13	0.14	0.08	-0.02	0.30	0.09
DescriptiveNorms_11 → InjunctiveNorms_13	0.01	0.07	-0.13	0.16	0.86
DescriptiveNorms_11 → DescriptiveNorms_13	0.31	0.08	0.15	0.46	0.00
DescriptiveNorms_11 → FactualBeliefs_13	0.11	0.08	-0.05	0.28	0.17
DescriptiveNorms_11 → PersonalBeliefs_13	0.07	0.08	-0.08	0.23	0.34
DescriptiveNorms_13 → MaskWearing_14	0.15	0.07	0.01	0.29	0.03
DescriptiveNorms_13 → InjunctiveNorms_14	0.21	0.06	0.09	0.33	0.00
DescriptiveNorms_13 → DescriptiveNorms_14	0.41	0.06	0.29	0.53	0.00
DescriptiveNorms_13 → FactualBeliefs_14	0.04	0.07	-0.09	0.17	0.54
DescriptiveNorms_13 → PersonalBeliefs_14	0.01	0.06	-0.10	0.12	0.85
DescriptiveNorms_14 → MaskWearing_15	0.09	0.09	-0.08	0.26	0.31
DescriptiveNorms_14 → InjunctiveNorms_15	-0.03	0.08	-0.18	0.11	0.65
DescriptiveNorms_14 → DescriptiveNorms_15	0.34	0.07	0.20	0.47	0.00

Table S5 continued

Parameter	Estimate	SE	2.5%	97.5%	p
DescriptiveNorms_14 → FactualBeliefs_15	0.13	0.09	-0.04	0.30	0.13
DescriptiveNorms_14 → PersonalBeliefs_15	0.07	0.08	-0.09	0.24	0.38
DescriptiveNorms_15 → MaskWearing_16	0.03	0.09	-0.14	0.20	0.69
DescriptiveNorms_15 → InjunctiveNorms_16	0.21	0.07	0.06	0.35	0.00
DescriptiveNorms_15 → DescriptiveNorms_16	0.25	0.06	0.13	0.37	0.00
DescriptiveNorms_15 → FactualBeliefs_16	0.20	0.09	0.02	0.38	0.03
DescriptiveNorms_15 → PersonalBeliefs_16	0.03	0.08	-0.13	0.18	0.72
DescriptiveNorms_16 → MaskWearing_17	0.12	0.12	-0.12	0.36	0.33
DescriptiveNorms_16 → InjunctiveNorms_17	0.22	0.08	0.07	0.37	0.01
DescriptiveNorms_16 → DescriptiveNorms_17	0.47	0.07	0.34	0.60	0.00
DescriptiveNorms_16 → FactualBeliefs_17	0.14	0.13	-0.10	0.39	0.26
DescriptiveNorms_16 → PersonalBeliefs_17	0.08	0.11	-0.13	0.30	0.45
DescriptiveNorms_17 → MaskWearing_18	0.03	0.12	-0.20	0.26	0.81
DescriptiveNorms_17 → InjunctiveNorms_18	0.28	0.07	0.14	0.42	0.00
DescriptiveNorms_17 → DescriptiveNorms_18	0.41	0.07	0.29	0.54	0.00
DescriptiveNorms_17 → FactualBeliefs_18	0.16	0.13	-0.09	0.41	0.22
DescriptiveNorms_17 → PersonalBeliefs_18	0.13	0.11	-0.09	0.36	0.24
FactualBeliefs_02 → MaskWearing_05	0.07	0.10	-0.13	0.26	0.50
FactualBeliefs_02 → InjunctiveNorms_05	-0.15	0.08	-0.30	0.01	0.06
FactualBeliefs_02 → DescriptiveNorms_05	-0.04	0.08	-0.20	0.12	0.62
FactualBeliefs_02 → FactualBeliefs_05	0.20	0.07	0.05	0.34	0.01
FactualBeliefs_02 → PersonalBeliefs_05	-0.09	0.07	-0.22	0.05	0.20
FactualBeliefs_05 → MaskWearing_09	0.29	0.12	0.05	0.53	0.02
FactualBeliefs_05 → InjunctiveNorms_09	-0.06	0.11	-0.27	0.16	0.60
FactualBeliefs_05 → DescriptiveNorms_09	-0.06	0.12	-0.29	0.17	0.60
FactualBeliefs_05 → FactualBeliefs_09	0.21	0.14	-0.05	0.48	0.12
FactualBeliefs_05 → PersonalBeliefs_09	0.03	0.13	-0.21	0.28	0.80
FactualBeliefs_09 → MaskWearing_11	0.16	0.09	-0.02	0.35	0.08
FactualBeliefs_09 → InjunctiveNorms_11	0.03	0.07	-0.11	0.18	0.63
FactualBeliefs_09 → DescriptiveNorms_11	0.08	0.07	-0.06	0.21	0.25

Table S5 continued

Parameter	Estimate	SE	2.5%	97.5%	p
FactualBeliefs_09 → FactualBeliefs_11	0.35	0.09	0.17	0.53	0.00
FactualBeliefs_09 → PersonalBeliefs_11	0.15	0.08	0.01	0.30	0.04
FactualBeliefs_11 → MaskWearing_13	0.12	0.07	-0.01	0.26	0.07
FactualBeliefs_11 → InjunctiveNorms_13	0.03	0.06	-0.09	0.15	0.59
FactualBeliefs_11 → DescriptiveNorms_13	-0.09	0.06	-0.22	0.03	0.14
FactualBeliefs_11 → FactualBeliefs_13	0.13	0.07	-0.01	0.26	0.06
FactualBeliefs_11 → PersonalBeliefs_13	0.10	0.06	-0.03	0.22	0.12
FactualBeliefs_13 → MaskWearing_14	0.03	0.08	-0.13	0.19	0.71
FactualBeliefs_13 → InjunctiveNorms_14	0.05	0.07	-0.09	0.18	0.49
FactualBeliefs_13 → DescriptiveNorms_14	0.00	0.07	-0.14	0.13	0.96
FactualBeliefs_13 → FactualBeliefs_14	0.27	0.08	0.12	0.42	0.00
FactualBeliefs_13 → PersonalBeliefs_14	0.16	0.07	0.03	0.29	0.02
FactualBeliefs_14 → MaskWearing_15	0.30	0.08	0.14	0.45	0.00
FactualBeliefs_14 → InjunctiveNorms_15	-0.03	0.07	-0.17	0.11	0.67
FactualBeliefs_14 → DescriptiveNorms_15	0.17	0.07	0.04	0.30	0.01
FactualBeliefs_14 → FactualBeliefs_15	0.53	0.08	0.37	0.68	0.00
FactualBeliefs_14 → PersonalBeliefs_15	0.34	0.08	0.18	0.49	0.00
FactualBeliefs_15 → MaskWearing_16	0.08	0.09	-0.09	0.26	0.36
FactualBeliefs_15 → InjunctiveNorms_16	0.08	0.08	-0.06	0.23	0.27
FactualBeliefs_15 → DescriptiveNorms_16	0.09	0.07	-0.04	0.22	0.19
FactualBeliefs_15 → FactualBeliefs_16	0.42	0.09	0.23	0.61	0.00
FactualBeliefs_15 → PersonalBeliefs_16	0.12	0.08	-0.04	0.29	0.13
FactualBeliefs_16 → MaskWearing_17	0.18	0.09	0.01	0.35	0.04
FactualBeliefs_16 → InjunctiveNorms_17	0.02	0.06	-0.09	0.13	0.78
FactualBeliefs_16 → DescriptiveNorms_17	-0.01	0.05	-0.11	0.09	0.82
FactualBeliefs_16 → FactualBeliefs_17	0.24	0.09	0.06	0.42	0.01
FactualBeliefs_16 → PersonalBeliefs_17	0.08	0.08	-0.08	0.24	0.33
FactualBeliefs_17 → MaskWearing_18	0.08	0.08	-0.08	0.23	0.34
FactualBeliefs_17 → InjunctiveNorms_18	-0.05	0.05	-0.15	0.04	0.26
FactualBeliefs_17 → DescriptiveNorms_18	0.04	0.04	-0.05	0.13	0.38

Table S5 continued

Parameter	Estimate	SE	2.5%	97.5%	p
FactualBeliefs_17 → FactualBeliefs_18	0.36	0.09	0.19	0.53	0.00
FactualBeliefs_17 → PersonalBeliefs_18	0.40	0.08	0.25	0.55	0.00
PersonalBeliefs_02 → MaskWearing_05	0.06	0.11	-0.16	0.28	0.58
PersonalBeliefs_02 → InjunctiveNorms_05	0.09	0.09	-0.09	0.26	0.33
PersonalBeliefs_02 → DescriptiveNorms_05	0.07	0.09	-0.11	0.24	0.45
PersonalBeliefs_02 → FactualBeliefs_05	0.16	0.08	-0.01	0.32	0.06
PersonalBeliefs_02 → PersonalBeliefs_05	0.41	0.08	0.25	0.57	0.00
PersonalBeliefs_05 → MaskWearing_09	-0.45	0.13	-0.70	-0.19	0.00
PersonalBeliefs_05 → InjunctiveNorms_09	-0.26	0.12	-0.49	-0.03	0.03
PersonalBeliefs_05 → DescriptiveNorms_09	-0.09	0.13	-0.33	0.16	0.50
PersonalBeliefs_05 → FactualBeliefs_09	-0.40	0.14	-0.68	-0.12	0.00
PersonalBeliefs_05 → PersonalBeliefs_09	-0.34	0.13	-0.60	-0.07	0.01
PersonalBeliefs_09 → MaskWearing_11	0.08	0.10	-0.12	0.27	0.45
PersonalBeliefs_09 → InjunctiveNorms_11	0.06	0.08	-0.09	0.22	0.44
PersonalBeliefs_09 → DescriptiveNorms_11	0.04	0.07	-0.11	0.18	0.60
PersonalBeliefs_09 → FactualBeliefs_11	0.10	0.10	-0.10	0.29	0.32
PersonalBeliefs_09 → PersonalBeliefs_11	0.19	0.08	0.02	0.35	0.02
PersonalBeliefs_11 → MaskWearing_13	0.06	0.08	-0.09	0.21	0.41
PersonalBeliefs_11 → InjunctiveNorms_13	0.07	0.07	-0.06	0.19	0.33
PersonalBeliefs_11 → DescriptiveNorms_13	0.11	0.07	-0.03	0.25	0.13
PersonalBeliefs_11 → FactualBeliefs_13	0.18	0.07	0.03	0.33	0.02
PersonalBeliefs_11 → PersonalBeliefs_13	0.17	0.07	0.04	0.31	0.01
PersonalBeliefs_13 → MaskWearing_14	0.27	0.09	0.09	0.44	0.00
PersonalBeliefs_13 → InjunctiveNorms_14	-0.10	0.08	-0.24	0.05	0.20
PersonalBeliefs_13 → DescriptiveNorms_14	-0.02	0.08	-0.17	0.13	0.80
PersonalBeliefs_13 → FactualBeliefs_14	0.36	0.08	0.20	0.53	0.00
PersonalBeliefs_13 → PersonalBeliefs_14	0.44	0.07	0.29	0.58	0.00
PersonalBeliefs_14 → MaskWearing_15	-0.06	0.09	-0.24	0.12	0.53
PersonalBeliefs_14 → InjunctiveNorms_15	0.12	0.08	-0.05	0.28	0.17
PersonalBeliefs_14 → DescriptiveNorms_15	-0.12	0.07	-0.26	0.03	0.12

Table S5 continued

Parameter	Estimate	SE	2.5%	97.5%	p
PersonalBeliefs_14 → FactualBeliefs_15	0.01	0.09	-0.17	0.19	0.89
PersonalBeliefs_14 → PersonalBeliefs_15	0.14	0.09	-0.04	0.32	0.14
PersonalBeliefs_15 → MaskWearing_16	0.09	0.09	-0.08	0.27	0.31
PersonalBeliefs_15 → InjunctiveNorms_16	0.05	0.08	-0.10	0.20	0.52
PersonalBeliefs_15 → DescriptiveNorms_16	0.10	0.07	-0.03	0.23	0.11
PersonalBeliefs_15 → FactualBeliefs_16	0.13	0.10	-0.06	0.32	0.17
PersonalBeliefs_15 → PersonalBeliefs_16	0.41	0.08	0.24	0.57	0.00
PersonalBeliefs_16 → MaskWearing_17	-0.02	0.09	-0.20	0.16	0.83
PersonalBeliefs_16 → InjunctiveNorms_17	0.00	0.06	-0.12	0.12	0.98
PersonalBeliefs_16 → DescriptiveNorms_17	-0.02	0.05	-0.12	0.08	0.73
PersonalBeliefs_16 → FactualBeliefs_17	0.28	0.10	0.08	0.47	0.00
PersonalBeliefs_16 → PersonalBeliefs_17	0.58	0.08	0.42	0.75	0.00
PersonalBeliefs_17 → MaskWearing_18	0.09	0.08	-0.07	0.24	0.26
PersonalBeliefs_17 → InjunctiveNorms_18	0.00	0.05	-0.10	0.09	0.96
PersonalBeliefs_17 → DescriptiveNorms_18	-0.04	0.04	-0.12	0.05	0.41
PersonalBeliefs_17 → FactualBeliefs_18	0.16	0.09	-0.01	0.33	0.07
PersonalBeliefs_17 → PersonalBeliefs_18	0.10	0.08	-0.05	0.25	0.21

Table S6

Unstandardized autoregressive and cross-lagged parameters from time-varying random-intercept cross-lagged panel model (with factual beliefs and personal normative beliefs removed). Variable name suffixes indicate time points. Arrows indicate the direction of prediction.

Parameter	Estimate	SE	2.5%	97.5%	p
MaskWearing_02 → MaskWearing_05	0.25	0.06	0.13	0.37	0.00
MaskWearing_02 → InjunctiveNorms_05	0.04	0.05	-0.06	0.13	0.46
MaskWearing_02 → DescriptiveNorms_05	0.03	0.05	-0.07	0.12	0.60
MaskWearing_05 → MaskWearing_09	0.15	0.07	0.03	0.28	0.02
MaskWearing_05 → InjunctiveNorms_09	0.09	0.06	-0.02	0.21	0.11
MaskWearing_05 → DescriptiveNorms_09	-0.03	0.06	-0.15	0.09	0.61
MaskWearing_09 → MaskWearing_11	-0.08	0.08	-0.24	0.08	0.31
MaskWearing_09 → InjunctiveNorms_11	0.03	0.06	-0.09	0.15	0.61
MaskWearing_09 → DescriptiveNorms_11	0.09	0.06	-0.02	0.21	0.11
MaskWearing_11 → MaskWearing_13	0.07	0.06	-0.04	0.18	0.22
MaskWearing_11 → InjunctiveNorms_13	0.04	0.05	-0.06	0.13	0.44
MaskWearing_11 → DescriptiveNorms_13	0.02	0.05	-0.08	0.12	0.71
MaskWearing_13 → MaskWearing_14	0.20	0.07	0.08	0.33	0.00
MaskWearing_13 → InjunctiveNorms_14	0.05	0.05	-0.06	0.16	0.36
MaskWearing_13 → DescriptiveNorms_14	0.08	0.06	-0.03	0.19	0.13
MaskWearing_14 → MaskWearing_15	0.25	0.07	0.12	0.38	0.00
MaskWearing_14 → InjunctiveNorms_15	0.05	0.06	-0.06	0.16	0.38
MaskWearing_14 → DescriptiveNorms_15	0.09	0.05	-0.01	0.19	0.07
MaskWearing_15 → MaskWearing_16	0.32	0.07	0.18	0.46	0.00
MaskWearing_15 → InjunctiveNorms_16	0.04	0.06	-0.08	0.16	0.52
MaskWearing_15 → DescriptiveNorms_16	0.05	0.05	-0.06	0.15	0.36
MaskWearing_16 → MaskWearing_17	0.42	0.07	0.29	0.56	0.00
MaskWearing_16 → InjunctiveNorms_17	0.00	0.04	-0.08	0.09	0.98
MaskWearing_16 → DescriptiveNorms_17	0.08	0.04	0.01	0.15	0.03
MaskWearing_17 → MaskWearing_18	0.43	0.05	0.33	0.54	0.00
MaskWearing_17 → InjunctiveNorms_18	-0.08	0.03	-0.15	-0.01	0.02
MaskWearing_17 → DescriptiveNorms_18	0.03	0.03	-0.03	0.09	0.37

Table S6 continued

Parameter	Estimate	SE	2.5%	97.5%	p
InjunctiveNorms_02 → MaskWearing_05	0.01	0.07	-0.13	0.15	0.92
InjunctiveNorms_02 → InjunctiveNorms_05	0.31	0.06	0.20	0.43	0.00
InjunctiveNorms_02 → DescriptiveNorms_05	0.07	0.06	-0.04	0.19	0.22
InjunctiveNorms_05 → MaskWearing_09	-0.10	0.08	-0.26	0.06	0.23
InjunctiveNorms_05 → InjunctiveNorms_09	0.07	0.08	-0.08	0.22	0.38
InjunctiveNorms_05 → DescriptiveNorms_09	0.00	0.08	-0.15	0.16	0.96
InjunctiveNorms_09 → MaskWearing_11	0.10	0.10	-0.09	0.29	0.29
InjunctiveNorms_09 → InjunctiveNorms_11	0.14	0.08	-0.01	0.29	0.07
InjunctiveNorms_09 → DescriptiveNorms_11	-0.01	0.07	-0.15	0.13	0.92
InjunctiveNorms_11 → MaskWearing_13	0.02	0.07	-0.12	0.17	0.75
InjunctiveNorms_11 → InjunctiveNorms_13	0.32	0.07	0.19	0.45	0.00
InjunctiveNorms_11 → DescriptiveNorms_13	0.22	0.07	0.08	0.36	0.00
InjunctiveNorms_13 → MaskWearing_14	0.00	0.08	-0.16	0.16	0.97
InjunctiveNorms_13 → InjunctiveNorms_14	0.39	0.07	0.26	0.53	0.00
InjunctiveNorms_13 → DescriptiveNorms_14	0.16	0.07	0.02	0.29	0.02
InjunctiveNorms_14 → MaskWearing_15	0.06	0.08	-0.11	0.22	0.50
InjunctiveNorms_14 → InjunctiveNorms_15	0.43	0.07	0.29	0.58	0.00
InjunctiveNorms_14 → DescriptiveNorms_15	0.30	0.07	0.17	0.43	0.00
InjunctiveNorms_15 → MaskWearing_16	0.22	0.09	0.05	0.39	0.01
InjunctiveNorms_15 → InjunctiveNorms_16	0.20	0.07	0.06	0.35	0.01
InjunctiveNorms_15 → DescriptiveNorms_16	0.08	0.06	-0.05	0.21	0.22
InjunctiveNorms_16 → MaskWearing_17	0.03	0.10	-0.17	0.22	0.80
InjunctiveNorms_16 → InjunctiveNorms_17	0.32	0.06	0.19	0.44	0.00
InjunctiveNorms_16 → DescriptiveNorms_17	0.10	0.05	0.00	0.21	0.06
InjunctiveNorms_17 → MaskWearing_18	0.01	0.10	-0.20	0.21	0.93
InjunctiveNorms_17 → InjunctiveNorms_18	0.37	0.06	0.24	0.49	0.00
InjunctiveNorms_17 → DescriptiveNorms_18	0.19	0.06	0.08	0.30	0.00
DescriptiveNorms_02 → MaskWearing_05	0.19	0.07	0.05	0.33	0.01
DescriptiveNorms_02 → InjunctiveNorms_05	0.14	0.06	0.03	0.26	0.01
DescriptiveNorms_02 → DescriptiveNorms_05	0.40	0.06	0.29	0.52	0.00

Table S6 continued

Parameter	Estimate	SE	2.5%	97.5%	p
DescriptiveNorms_05 → MaskWearing_09	0.21	0.08	0.05	0.36	0.01
DescriptiveNorms_05 → InjunctiveNorms_09	0.19	0.07	0.05	0.33	0.01
DescriptiveNorms_05 → DescriptiveNorms_09	0.27	0.08	0.12	0.42	0.00
DescriptiveNorms_09 → MaskWearing_11	0.11	0.09	-0.06	0.28	0.22
DescriptiveNorms_09 → InjunctiveNorms_11	0.20	0.07	0.06	0.33	0.00
DescriptiveNorms_09 → DescriptiveNorms_11	0.26	0.07	0.13	0.38	0.00
DescriptiveNorms_11 → MaskWearing_13	0.18	0.08	0.03	0.34	0.02
DescriptiveNorms_11 → InjunctiveNorms_13	0.03	0.07	-0.10	0.17	0.63
DescriptiveNorms_11 → DescriptiveNorms_13	0.29	0.08	0.14	0.44	0.00
DescriptiveNorms_13 → MaskWearing_14	0.17	0.07	0.03	0.31	0.02
DescriptiveNorms_13 → InjunctiveNorms_14	0.21	0.06	0.09	0.33	0.00
DescriptiveNorms_13 → DescriptiveNorms_14	0.41	0.06	0.29	0.53	0.00
DescriptiveNorms_14 → MaskWearing_15	0.13	0.09	-0.04	0.30	0.15
DescriptiveNorms_14 → InjunctiveNorms_15	-0.01	0.08	-0.15	0.14	0.94
DescriptiveNorms_14 → DescriptiveNorms_15	0.34	0.07	0.21	0.47	0.00
DescriptiveNorms_15 → MaskWearing_16	0.07	0.09	-0.10	0.24	0.44
DescriptiveNorms_15 → InjunctiveNorms_16	0.25	0.07	0.11	0.40	0.00
DescriptiveNorms_15 → DescriptiveNorms_16	0.28	0.06	0.15	0.41	0.00
DescriptiveNorms_16 → MaskWearing_17	0.18	0.12	-0.06	0.41	0.14
DescriptiveNorms_16 → InjunctiveNorms_17	0.24	0.08	0.09	0.39	0.00
DescriptiveNorms_16 → DescriptiveNorms_17	0.45	0.06	0.33	0.58	0.00
DescriptiveNorms_17 → MaskWearing_18	0.10	0.12	-0.12	0.33	0.37
DescriptiveNorms_17 → InjunctiveNorms_18	0.28	0.07	0.14	0.42	0.00
DescriptiveNorms_17 → DescriptiveNorms_18	0.42	0.06	0.30	0.55	0.00

Table S7

Unstandardized autoregressive and cross-lagged parameters from time-varying random-intercept cross-lagged panel model (with factual beliefs, personal normative beliefs, and all exogenous covariates removed). Variable name suffixes indicate time points. Arrows indicate the direction of prediction.

Parameter	Estimate	SE	2.5%	97.5%	p
MaskWearing_02 → MaskWearing_05	0.24	0.06	0.12	0.36	0.00
MaskWearing_02 → InjunctiveNorms_05	0.08	0.05	-0.02	0.18	0.13
MaskWearing_02 → DescriptiveNorms_05	0.04	0.05	-0.06	0.14	0.46
MaskWearing_05 → MaskWearing_09	0.16	0.07	0.03	0.29	0.02
MaskWearing_05 → InjunctiveNorms_09	0.09	0.06	-0.03	0.20	0.15
MaskWearing_05 → DescriptiveNorms_09	0.00	0.06	-0.13	0.12	0.98
MaskWearing_09 → MaskWearing_11	-0.07	0.08	-0.23	0.09	0.38
MaskWearing_09 → InjunctiveNorms_11	0.03	0.06	-0.09	0.15	0.58
MaskWearing_09 → DescriptiveNorms_11	0.13	0.06	0.02	0.24	0.02
MaskWearing_11 → MaskWearing_13	0.11	0.05	0.00	0.22	0.05
MaskWearing_11 → InjunctiveNorms_13	0.08	0.05	-0.01	0.18	0.07
MaskWearing_11 → DescriptiveNorms_13	0.07	0.05	-0.03	0.16	0.18
MaskWearing_13 → MaskWearing_14	0.23	0.06	0.11	0.36	0.00
MaskWearing_13 → InjunctiveNorms_14	0.06	0.05	-0.04	0.17	0.23
MaskWearing_13 → DescriptiveNorms_14	0.13	0.05	0.02	0.24	0.02
MaskWearing_14 → MaskWearing_15	0.27	0.07	0.13	0.40	0.00
MaskWearing_14 → InjunctiveNorms_15	0.05	0.06	-0.06	0.16	0.36
MaskWearing_14 → DescriptiveNorms_15	0.12	0.05	0.02	0.22	0.02
MaskWearing_15 → MaskWearing_16	0.36	0.07	0.22	0.49	0.00
MaskWearing_15 → InjunctiveNorms_16	0.06	0.06	-0.05	0.18	0.28
MaskWearing_15 → DescriptiveNorms_16	0.09	0.05	-0.01	0.19	0.10
MaskWearing_16 → MaskWearing_17	0.42	0.06	0.29	0.54	0.00
MaskWearing_16 → InjunctiveNorms_17	-0.02	0.04	-0.10	0.06	0.60
MaskWearing_16 → DescriptiveNorms_17	0.08	0.04	0.01	0.15	0.03
MaskWearing_17 → MaskWearing_18	0.46	0.05	0.35	0.56	0.00
MaskWearing_17 → InjunctiveNorms_18	-0.06	0.03	-0.12	0.00	0.07
MaskWearing_17 → DescriptiveNorms_18	0.02	0.03	-0.03	0.08	0.42

Table S7 continued

Parameter	Estimate	SE	2.5%	97.5%	p
InjunctiveNorms_02 → MaskWearing_05	-0.02	0.07	-0.17	0.12	0.73
InjunctiveNorms_02 → InjunctiveNorms_05	0.29	0.06	0.18	0.41	0.00
InjunctiveNorms_02 → DescriptiveNorms_05	0.08	0.06	-0.04	0.19	0.20
InjunctiveNorms_05 → MaskWearing_09	-0.13	0.08	-0.28	0.03	0.11
InjunctiveNorms_05 → InjunctiveNorms_09	0.07	0.08	-0.07	0.22	0.32
InjunctiveNorms_05 → DescriptiveNorms_09	-0.03	0.08	-0.18	0.13	0.74
InjunctiveNorms_09 → MaskWearing_11	0.16	0.10	-0.03	0.35	0.10
InjunctiveNorms_09 → InjunctiveNorms_11	0.16	0.07	0.01	0.30	0.03
InjunctiveNorms_09 → DescriptiveNorms_11	0.01	0.07	-0.12	0.15	0.84
InjunctiveNorms_11 → MaskWearing_13	0.05	0.07	-0.09	0.20	0.48
InjunctiveNorms_11 → InjunctiveNorms_13	0.31	0.07	0.18	0.44	0.00
InjunctiveNorms_11 → DescriptiveNorms_13	0.22	0.07	0.09	0.36	0.00
InjunctiveNorms_13 → MaskWearing_14	0.01	0.08	-0.14	0.17	0.87
InjunctiveNorms_13 → InjunctiveNorms_14	0.45	0.07	0.32	0.58	0.00
InjunctiveNorms_13 → DescriptiveNorms_14	0.21	0.07	0.07	0.34	0.00
InjunctiveNorms_14 → MaskWearing_15	0.11	0.08	-0.05	0.27	0.19
InjunctiveNorms_14 → InjunctiveNorms_15	0.45	0.07	0.32	0.59	0.00
InjunctiveNorms_14 → DescriptiveNorms_15	0.33	0.06	0.20	0.45	0.00
InjunctiveNorms_15 → MaskWearing_16	0.22	0.09	0.05	0.39	0.01
InjunctiveNorms_15 → InjunctiveNorms_16	0.23	0.07	0.09	0.38	0.00
InjunctiveNorms_15 → DescriptiveNorms_16	0.12	0.06	-0.01	0.25	0.06
InjunctiveNorms_16 → MaskWearing_17	0.02	0.10	-0.17	0.21	0.83
InjunctiveNorms_16 → InjunctiveNorms_17	0.34	0.06	0.22	0.46	0.00
InjunctiveNorms_16 → DescriptiveNorms_17	0.11	0.05	0.00	0.21	0.05
InjunctiveNorms_17 → MaskWearing_18	0.02	0.10	-0.18	0.21	0.87
InjunctiveNorms_17 → InjunctiveNorms_18	0.42	0.06	0.31	0.54	0.00
InjunctiveNorms_17 → DescriptiveNorms_18	0.19	0.05	0.08	0.29	0.00
DescriptiveNorms_02 → MaskWearing_05	0.22	0.07	0.09	0.36	0.00
DescriptiveNorms_02 → InjunctiveNorms_05	0.16	0.06	0.04	0.27	0.01
DescriptiveNorms_02 → DescriptiveNorms_05	0.40	0.06	0.29	0.51	0.00

Table S7 continued

Parameter	Estimate	SE	2.5%	97.5%	p
DescriptiveNorms_05 → MaskWearing_09	0.23	0.08	0.07	0.38	0.00
DescriptiveNorms_05 → InjunctiveNorms_09	0.20	0.07	0.06	0.35	0.00
DescriptiveNorms_05 → DescriptiveNorms_09	0.30	0.08	0.14	0.45	0.00
DescriptiveNorms_09 → MaskWearing_11	0.14	0.09	-0.04	0.31	0.12
DescriptiveNorms_09 → InjunctiveNorms_11	0.23	0.07	0.10	0.36	0.00
DescriptiveNorms_09 → DescriptiveNorms_11	0.29	0.06	0.16	0.41	0.00
DescriptiveNorms_11 → MaskWearing_13	0.24	0.08	0.08	0.39	0.00
DescriptiveNorms_11 → InjunctiveNorms_13	0.07	0.07	-0.06	0.21	0.29
DescriptiveNorms_11 → DescriptiveNorms_13	0.30	0.07	0.16	0.44	0.00
DescriptiveNorms_13 → MaskWearing_14	0.18	0.07	0.04	0.32	0.01
DescriptiveNorms_13 → InjunctiveNorms_14	0.21	0.06	0.09	0.33	0.00
DescriptiveNorms_13 → DescriptiveNorms_14	0.38	0.06	0.26	0.51	0.00
DescriptiveNorms_14 → MaskWearing_15	0.13	0.09	-0.04	0.30	0.14
DescriptiveNorms_14 → InjunctiveNorms_15	0.04	0.07	-0.10	0.18	0.61
DescriptiveNorms_14 → DescriptiveNorms_15	0.36	0.07	0.23	0.49	0.00
DescriptiveNorms_15 → MaskWearing_16	0.10	0.09	-0.06	0.27	0.22
DescriptiveNorms_15 → InjunctiveNorms_16	0.27	0.07	0.13	0.41	0.00
DescriptiveNorms_15 → DescriptiveNorms_16	0.29	0.06	0.17	0.42	0.00
DescriptiveNorms_16 → MaskWearing_17	0.16	0.12	-0.07	0.39	0.17
DescriptiveNorms_16 → InjunctiveNorms_17	0.26	0.07	0.12	0.40	0.00
DescriptiveNorms_16 → DescriptiveNorms_17	0.47	0.06	0.35	0.60	0.00
DescriptiveNorms_17 → MaskWearing_18	0.21	0.11	-0.01	0.42	0.06
DescriptiveNorms_17 → InjunctiveNorms_18	0.30	0.06	0.17	0.42	0.00
DescriptiveNorms_17 → DescriptiveNorms_18	0.46	0.06	0.34	0.58	0.00

Table S8

List of norm interpretation questions asked at Time 7. These questions were preceded by the following text: *"There may or may not be a difference between what people around you are doing and what they should be doing. You can learn about what people are doing and what they should be doing in different ways. For each of the following information sources, we want to know if you can learn from it what people are doing, what people should be doing, or both".* Participants answered all questions on a 7-point Likert scale, from (1) Not At All to (7) Very Strongly.

Interpretation	Item	Question	Mean	SD
Provides descriptive information	Descriptive	Does noticing the proportion of people in your area that wear a mask while doing recreational/social activities indoors (e.g., going to the gym, eating at a restaurant, attending a party) tell you what everyone is doing?	4.65	1.68
		Does noticing the proportion of people in your area that wear a mask while doing routine activities indoors (e.g., running errands, shopping, going to work) tell you what everyone is doing?	4.86	1.65
	Injunctive	Do mask-wearing rules encouraged in your area (e.g., by local or state government officials, businesses, etc.) tell you what everyone is doing?	4.35	1.89
		Does how often you see people that you respect and trust wearing a mask (e.g., on tv, news, etc.) tell you what everyone is doing?	4.16	1.88
Provides injunctive information	Descriptive	Does noticing the proportion of people in your area that wear a mask while doing recreational/social activities indoors (e.g., going to the gym, eating at a restaurant, attending a party) tell you what everyone should be doing?	5.09	1.90
		Does noticing the proportion of people in your area that wear a mask while doing routine activities indoors (e.g., running errands, shopping, going to work) tell you what everyone should be doing?	5.14	1.87
	Injunctive	Do mask-wearing rules encouraged in your area (e.g., by local or state government officials, businesses, etc.) tell you what everyone should be doing?	5.70	1.73
		Does how often you see people that you respect and trust wearing a mask (e.g., on tv, news, etc.) tell you what everyone should be doing?	5.38	1.75

Table S9

Pairwise comparisons from a multilevel model testing the construct validity of the four self-reported social norm items. Data were analysed in long format, with the self-report social norm item and the type of normative information provided (descriptive or injunctive) as predictors and a random intercept for participant. All pairwise comparisons account for multiple comparisons using Tukey adjustment.

Type	Pairwise contrast	Estimate	SE	p
Descriptive	MaskEncouraged - MaskRespect	0.19	0.09	0.11
Descriptive	MaskEncouraged - NeighborMask1	-0.51	0.09	0.00
Descriptive	MaskEncouraged - NeighborMask2	-0.30	0.09	0.00
Descriptive	MaskRespect - NeighborMask1	-0.70	0.09	0.00
Descriptive	MaskRespect - NeighborMask2	-0.49	0.09	0.00
Descriptive	NeighborMask1 - NeighborMask2	0.21	0.09	0.07
Injunctive	MaskEncouraged - MaskRespect	0.32	0.09	0.00
Injunctive	MaskEncouraged - NeighborMask1	0.55	0.09	0.00
Injunctive	MaskEncouraged - NeighborMask2	0.61	0.09	0.00
Injunctive	MaskRespect - NeighborMask1	0.24	0.09	0.03
Injunctive	MaskRespect - NeighborMask2	0.29	0.09	0.00
Injunctive	NeighborMask1 - NeighborMask2	0.05	0.09	0.93

Table S10

Unstandardized autoregressive and cross-lagged parameters from multi-group time-invariant random-intercept cross-lagged panel model, split by majority-Democrat and majority-Republican states. The model contains all covariates. Arrows indicate the direction of prediction.

Group	Parameter	Estimate	SE	2.5%	97.5%	p
Democrat	Mask wearing → Mask wearing	0.20	0.04	0.13	0.27	0.00
Democrat	Mask wearing → Injunctive norms	0.01	0.03	-0.04	0.06	0.81
Democrat	Mask wearing → Descriptive norms	0.01	0.03	-0.04	0.06	0.68
Democrat	Injunctive norms → Mask wearing	-0.02	0.04	-0.11	0.06	0.59
Democrat	Injunctive norms → Injunctive norms	0.32	0.04	0.24	0.39	0.00
Democrat	Injunctive norms → Descriptive norms	0.21	0.04	0.14	0.28	0.00
Democrat	Descriptive norms → Mask wearing	0.08	0.04	0.00	0.17	0.06
Democrat	Descriptive norms → Injunctive norms	0.24	0.03	0.17	0.30	0.00
Democrat	Descriptive norms → Descriptive norms	0.33	0.04	0.25	0.40	0.00
Republican	Mask wearing → Mask wearing	0.19	0.04	0.11	0.26	0.00
Republican	Mask wearing → Injunctive norms	0.05	0.03	-0.01	0.10	0.10
Republican	Mask wearing → Descriptive norms	0.07	0.03	0.02	0.12	0.01
Republican	Injunctive norms → Mask wearing	0.00	0.04	-0.08	0.09	0.91
Republican	Injunctive norms → Injunctive norms	0.24	0.04	0.16	0.31	0.00
Republican	Injunctive norms → Descriptive norms	0.05	0.03	-0.01	0.11	0.12
Republican	Descriptive norms → Mask wearing	0.14	0.05	0.05	0.24	0.00
Republican	Descriptive norms → Injunctive norms	0.11	0.04	0.04	0.19	0.00
Republican	Descriptive norms → Descriptive norms	0.32	0.04	0.24	0.39	0.00

Supplementary References

Pearl, J. (1995). Causal diagrams for empirical research. *Biometrika*, *82*(4), 669-688.
